# SpeCollate: Deep cross-modal similarity network for mass spectrometry data based peptide deductions

**DOI:** 10.1371/journal.pone.0259349

**Published:** 2021-10-29

**Authors:** Muhammad Usman Tariq, Fahad Saeed

**Affiliations:** School of Computing & Information Sciences, Florida International University, Miami, FL, United States of America; Swiss Institute of Bioinformatics, SWITZERLAND

## Abstract

Historically, the database search algorithms have been the de facto standard for inferring peptides from mass spectrometry (MS) data. Database search algorithms deduce peptides by transforming theoretical peptides into theoretical spectra and matching them to the experimental spectra. Heuristic similarity-scoring functions are used to match an experimental spectrum to a theoretical spectrum. However, the heuristic nature of the scoring functions and the *simple* transformation of the peptides into theoretical spectra, along with noisy mass spectra for the less abundant peptides, can introduce a cascade of inaccuracies. In this paper, we design and implement a Deep *Cross-Modal* Similarity Network called *SpeCollate*, which overcomes these inaccuracies by learning the similarity function between experimental spectra and peptides directly from the labeled MS data. SpeCollate transforms spectra and peptides into a shared Euclidean subspace by learning fixed size embeddings for both. Our proposed deep-learning network trains on sextuplets of positive and negative examples coupled with our custom-designed *SNAP-loss* function. Online hardest negative mining is used to select the appropriate negative examples for optimal training performance. We use 4.8 million sextuplets obtained from the NIST and MassIVE peptide libraries to train the network and demonstrate that for closed search, SpeCollate is able to perform better than Crux and MSFragger in terms of the number of peptide-spectrum matches (PSMs) and unique peptides identified under 1% FDR for real-world data. SpeCollate also identifies a large number of peptides not reported by either Crux or MSFragger. To the best of our knowledge, our proposed SpeCollate is the first deep-learning network that can determine the cross-modal similarity between peptides and mass-spectra for MS-based proteomics. We believe *SpeCollate* is significant progress towards developing machine-learning solutions for MS-based omics data analysis. SpeCollate is available at https://deepspecs.github.io/.

## 1 Introduction

**Fig 1:**
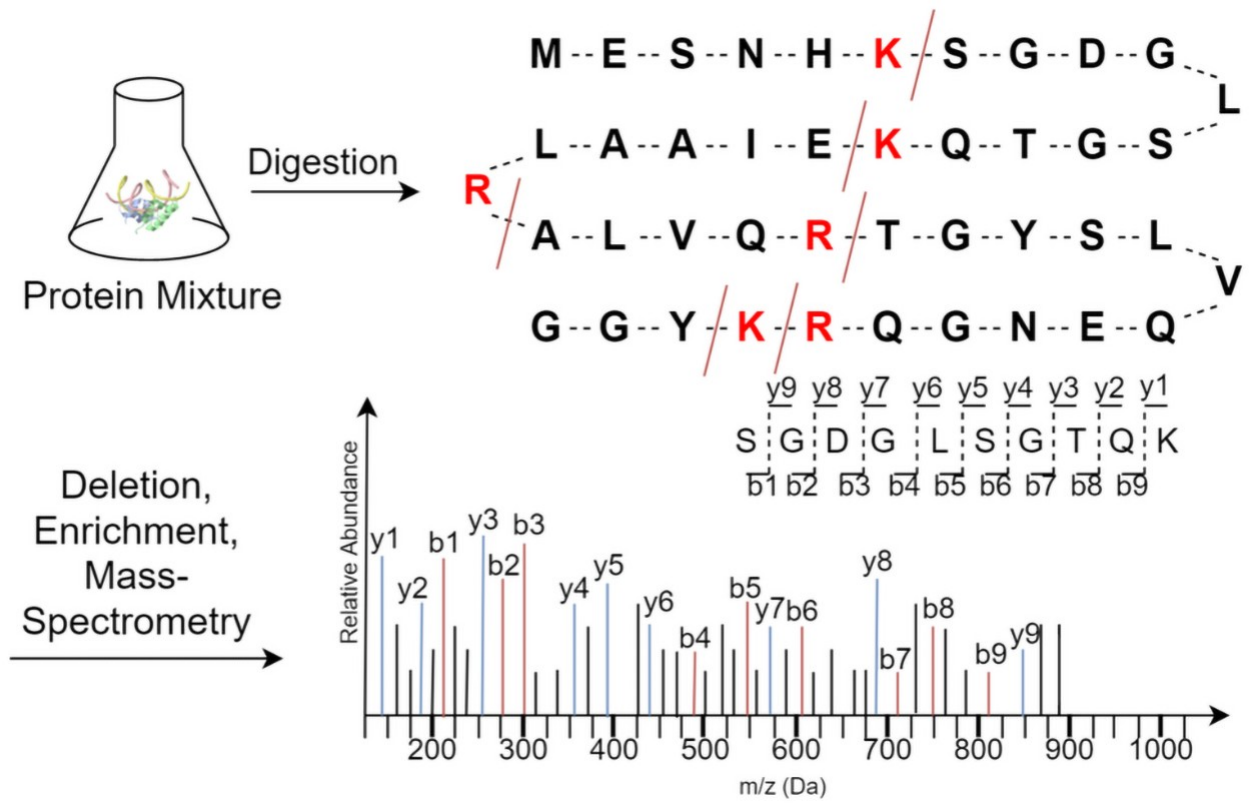
Process of generating MS/MS spectra from a protein mixture using mass-spectrometry analysis. Protein in the mixture are broken into peptides using the enzyme called trypsin which breaks the protein strings at K and R bases generating peptides of varying sizes. This peptide mixture is then refined and peptides are moved through mass-spectrometer which generates an MS/MS spectrum for each different peptide.

To date, mass spectrometry (MS) proteomics data is identified using database search algorithms purely based on numerical techniques (Fig 1). These numerical techniques operate by comparing the experimental spectra to the simulated spectra generated from theoretical peptides using a simple simulator [1–4]. The experimental spectra are matched against the theoretical ones using one of many available heuristic scoring-functions including *dot product* [5], *shared peak count* [6, 7], and *ion matches* [8]. Other peptide identification techniques such de novo algorithms [9–21] also deduce peptides directly from experimental spectra with varying degree of success.

**Fig 2:**
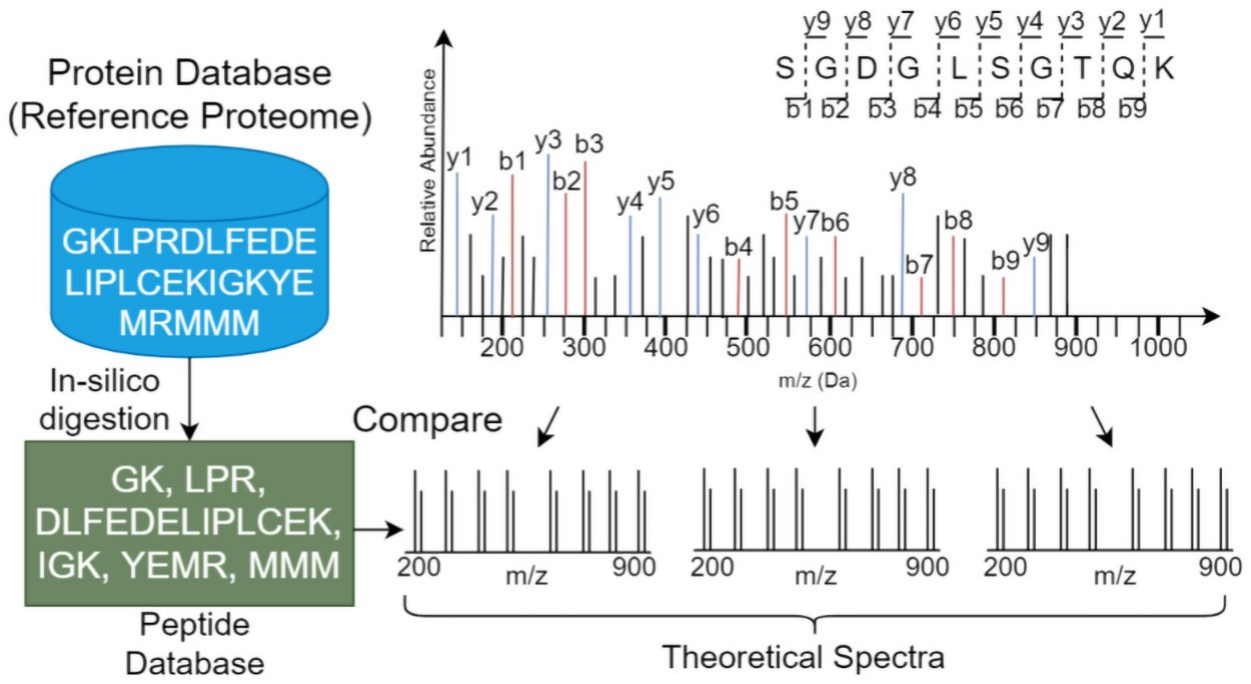
A generic proteomics flow. In-silico digestion of the protein database is performed to generate peptides. These peptides are then converted to the theoretical spectra and compared against the experimental spectra.

Currently, there is no single scoring function from database search techniques that can claim as the most accurate strategy. Substantial work has been carried out towards developing computational techniques for identification of peptides using database search [5–8] (See Fig 2 for a generic proteomics database search workflow), as well as de novo algorithms [9–21]. However, peptide identification problems are well-known and prevalent including but not limited to misidentifications or no identifications for peptides, statistical accuracy (FDR), and inconsistencies between different search engines [22]. Comparison across literature indicates decreased average accuracy of de novo algorithms (< 35%) [12] relative to database search algorithms (30–80%) [23]. Lack of quality assessment benchmarks makes the accuracy exhibited from these database search tools highly dependent on the data, indicating that further formal investigation and evaluation is warranted. Two major sources of heuristic errors that are introduced in the numerical database search algorithms are (1) how the peptide deduction takes place, i.e., simulation of the spectra (from peptides), and (2) the peptide spectrum match scoring-function.

Previous work suggests that numerical algorithms and the use of traditional ML algorithms may not be able to capture and integrate the multidimensional features of MS data [24]. However, deep learning methods [12, 13] may offer an improved approach for identifying peptides in noisy high-dimensional MS data and peptides that are very similar to each other [3]. Preliminary progress assessing deep learning methods in peptide deduction applied to MS data has yielded an average accuracy of 82–95% on selected data sets, but with limited precision (amino-acid level 72%) and recall (peptide level 39.24%) [13]. Prosit [2] incorporates theoretical spectral simulation in the database search pipeline by encoding the peptide sequences into a latent space and then decoding the embeddings to predict fragment ion intensities while Slider [25] uses deep convolutional neural networks to score experimental spectra against theoretical spectra. In [26], spectra for modified peptides are embedded close to the non-modified version in vectors of length 32, which enables them for fast lookup of the non-modified version for a given spectrum. These studies have shown that deep-learning models can be helpful in modeling MS proteomics data and improved accuracy from these peptide algorithms warrants further investigation into the applicability of sophisticated machine-learning strategies.

In this paper, we make two fundamental contributions in MS-based proteomics search: 1) We present the design and implementation of Deep Similarity Network, called *SpeCollate*, for peptide-spectrum similarity measure. The goal is to learn a fixed-sized embedding of variable length *experimental spectra*, and peptide strings so that spectrum and its corresponding peptide are projected close to each other in the shared embedding space. Our proposed network consists of two sub-networks, i.e., spectrum sub-network (SSN) consisting of two fully connected layers and peptide sub-network (PSN) consisting of one bi-directional LSTM followed by two fully connected layers. 2) We then formulate and implement a custom loss function, called *SNAP-loss* for training the proposed deep-similarity network SpeCollate. This training process takes sextuplets as input, consisting of a combination of positive, negative, and anchor spectra and peptides. We train or network for 200 epochs on a dataset of size ∼ 4.8 million sextuplets containing ∼ 1.8 million unique peptides. As SpeCollate generates charge-independent peptide embeddings, we demonstrate that it performs better than Crux and MSFragger in terms of the number of PSMs and peptides identified under 1% FDR. SpeCollate encodes *both* peptides, and spectra into a latent space which are then used for *direct* comparison and peptide deduction. To the best of our knowledge, the proposed SpeCollate is the first deep-learning network that can determine *cross-modal* similarity between peptides and mass-spectra for MS-based omics.

The rest of the paper is organized as follows: In section 2, we discuss different motivations for our work. In section 3, we discuss the major contributions of this work and discuss the design and implementation of SpeCollate, SNAP-loss, and GPU-based inference. In section 4, we describe experimental setup and present results. In section 5, we discuss the results, limitations of SpeCollate, and future prospects. Section 6 concludes this paper.

## 2 Background

In this section, we will discuss the background of proteomics database search, emphasizing the shortcomings of the existing methods and potential solutions for them that inspired the development of the current framework. The shortcomings, including limitations and oversights of the existing numerical techniques, bounded performance of spectral simulators, unoptimized scoring heuristics, and the opportunities made available by huge data repositories with labeled spectra, are discussed.

### 2.1 Space transition

**Fig 3:**
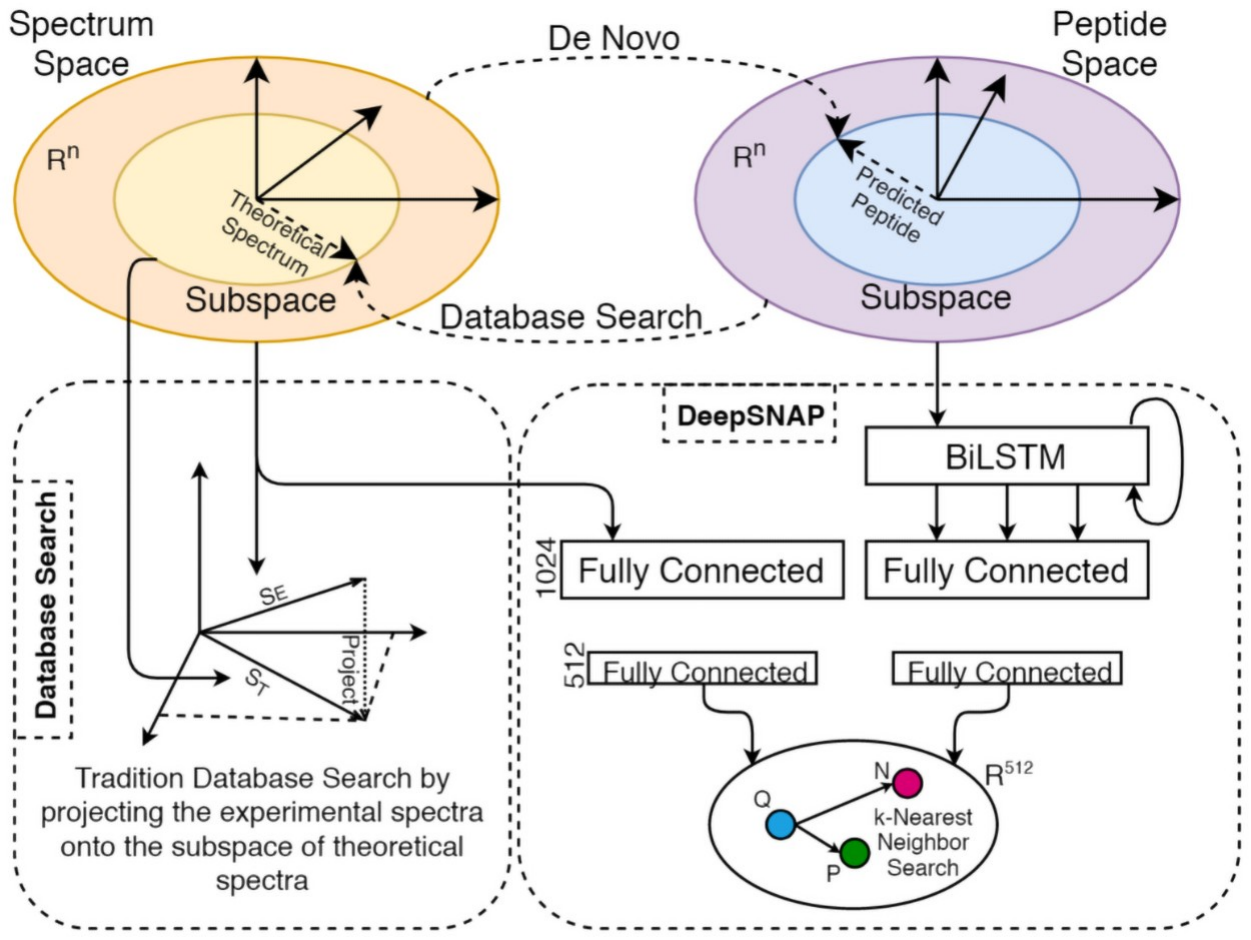
Space transition methods. De novo and database search, that try to transform one space to another. This is prone to error and uncertainty as a lot of information can be missed. On the contrary, SpeCollate learns same sized embeddings for both peptides and spectra by projecting them to a shared euclidean space.

Peptides and their corresponding MS/MS spectra lie in vastly distinct spaces. Peptides consist of a string of (typically) twenty alphabets (each representing an amino acid). In contrast, spectra are a series of floating-point numbers generated by a complex and stochastic fragmentation process. Transitioning in-between spaces can only approximate the counterpart projection as manifested by the existing techniques. De novo projects spectra onto a sub-peptide-space but with underwhelming accuracy as the spectra are mostly noisy and necessary information is missing. Similarly, in peptide-spectrum scoring methods, peptides are projected onto sub-spectral-space, and the similarity is measured by projecting the experimental spectra onto the same subspace (dot-product) for comparison as shown in Fig 3. Although the database search process is relatively more accurate, the output quality is contingent on the quality of the experimental and projected theoretical spectra. Therefore, we argue that a more flexible technique that can learn intermediate embeddings for both spectra and peptides could improve database search quality.

### 2.2 Spectral simulation

Most state-of-the-art database search tools provide a simulator to generate spectra containing b and y peaks (sometimes a, c, x, and z) from the theoretical peptides. Other simulators also provide options to generate peaks with different neutral losses (NH_3_, H_2_O), Immonium ions, and isotope ions [27]. These simulators incur numerous deficiencies due to the inherent complexities of the mass-spectrometry process, causing misidentification of multiple features. These include unaccounted peaks, missing peaks, falsely identified true and noisy peaks, peak intensities, neutral losses, isotopic peaks, and noise characteristics. As a result, the simulated spectra only manage to span a sub-space of experimental spectra (Fig 3). Recently, various deep learning-based simulators have mitigated the problems with the traditional simulation approaches to a certain extent. One of the main contributions of this paper is to directly match the experimental spectra and their corresponding theoretical peptide string by learning the embeddings in a shared subspace from huge sets of labeled data available, e.g., NIST and MassIVE.

### 2.3 Scoring heuristics

Although the simulators somewhat help improve the database search process, they only address half of the challenge, i.e., spectra simulation, while the scoring function is still the limiting factor. Presence or absence of specific peaks/ions can significantly impact peptide spectrum matching. In addition, the comparison of complex fragmentation spectra is not straightforward, and the outcome can vary among different scoring functions. SpeCollate can overcome this shortcoming by training on a wide variety of data to learn embeddings optimized for distance-based similarity. By processing data from different modalities using different types of networks, i.e., spectra using SSN and peptides using PSN, it can extract the valuable features needed for proper matching.

### 2.4 Learning from data

When learning a similarity (or scoring) function, ideally, we would like to retain all the features that improve the similarity measure and abolish the useless ones. SpeCollate approaches this solution by projecting both peptides and spectra onto a shared euclidean space. This is accomplished by learning embeddings of equal size for both spectra and peptides so that their similarity is directly proportional to L2 distance in the resultant euclidean space. Hence, addressing both above-mentioned fundamental problems by finding a middle ground between two extremes (De novo and database search) and simplifying the comparison. Using separate branches for spectra and peptides, SpeCollate can learn valuable features to assign spectra to their corresponding peptides confidently using the L2 distance-based similarity measure.

## 3 SpeCollate: Deep learning model for spectra-peptide similarity

This paper designs and implements a similarity network (SpeCollate) and the custom loss function (SNAP-loss) to learn a similarity function for peptide-spectrum matches. Similarity networks have been used for cross-modal retrieval of images and captions [28], face re/identification [29], and other problems [30, 31]. In proteomics, the use of similarity networks has been limited to spectral library search [26] and spectral clustering [32]. Our goal is to learn a fixed-sized embedding of variable length experimental spectra and peptide strings so that a given spectrum and its corresponding peptide are projected close to each other (in terms of L2 distance) in the shared subspace. We use L2 distance as the similarity matrix since it has been shown in the literature to work well for similarity ranking loss functions, e.g., triplet loss, and perform better than other similarity metrics, e.g., cosine similarity. We took inspiration from FaceNet [29] to select L2 distance as the similarity metric. The network consists of two sub-networks, i.e., SSN consisting of two fully connected layers and PSN consisting of one bi-directional LSTM followed by two fully connected layers. The training process takes two sets of data points as inputs, i.e., the sparse spectrum vectors and encoded peptide strings. The loss value is calculated by generating sextuplets, after each forward pass, consisting of a positive pair (*Q*, *P*), a negative pair (*Q*_*N*_, *P*_*N*_)_*Q*_ for *Q*, and a negative pair (*Q*_*N*_, *P*_*N*_)_*P*_ for *P* where *Q* is the anchor spectrum and *P* is the positive peptide. The negative pairs are selected via online hardest negative mining to make the training process more efficient and faster. In this process, the negative spectra and peptides closest to *Q* and *P* are selected for a given batch after each forward pass.

In this regard, this paper’s contributions are two folds: 1) Design and implementation of Deep Similarity Network, SpeCollate, for Proteomics Database Search and 2) Formulation and deployment of custom loss function SNAP-loss for training SpeCollate.

### 3.1 SpeCollate architecture

**Fig 4:**
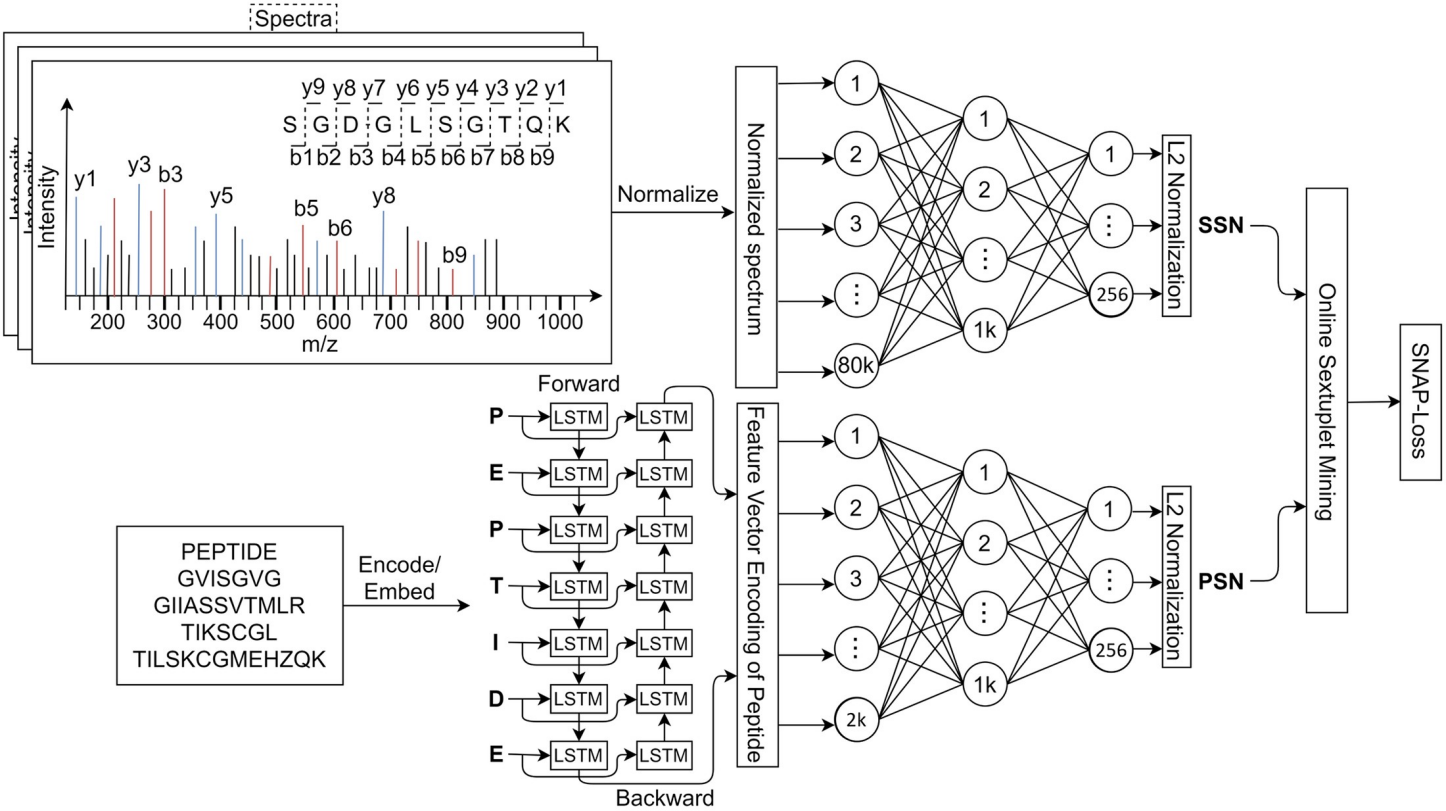
SpeCollate: Deep Similarity Network for proteomics. The spectra Q are passed to SSN in the form of sparse one-hot normalized representation. The positive and negative peptides (P, N) are passed to PSN one by one in both forward and backward direction.

SpeCollate consists of two sub-networks called spectral sub-network (SSN) and peptide sub-network (PSN), which process spectra and peptides respectively to generate embeddings of size 256. We empirically selected the embedding size of 256 as the smallest value without incurring any performance degradation. The complete SpeCollate network is shown in Fig 4.

#### 3.1.1 Spectral Sub-Network (SSN)

The SSN branch of the network processes spectra and embeds them onto the surface of a unit hyper-sphere in a euclidean subspace ${I\;R}^{256}$. SSN consists of two fully connected hidden layers of size 80, 000 × 1024 and 1024 × 256 respectively and a *L*2 normalization output layer. The input layer is of size 80, 000, which takes spectra as input in the form of sparse vectors with intensity values normalized to zero mean and unit variance and mass binning of 0.1 Da. Both hidden layers use rectified linear unit or ReLU as the activation function. A dropout mechanism with the probability value of 0.3 is used after the first hidden layer to avoid over-fitting.

#### 3.1.2 Peptide Sub-Network (PSN)

The PSN branch of SpeCollate processes the peptides and embeds them onto the surface of the same hyper-sphere in ${I\;R}^{256}$ enabling the direct comparison between spectra and peptides. The PSN consists of one bi-directional long short-term memory (Bi-LSTM) layer followed by two fully connected layers. An embedding layer is added before the Bi-LSTM layer to embed each amino acid character into a floating-point vector of size 256. We use a vocabulary of size 30 including 20 amino acids, 9 modifications, and a blank space. Bi-LSTM has the hidden dimension of 1024, and the outputs from both forward and backward passes are concatenated to get an output of a total length of 2048. This output is further fed to two fully connected layers of size 2048 × 1024 and 1024 × 256. ReLU activation function is used for the fully connected layers, and dropout with the probability of 0.3 is used after the Bi-LSTM and the first fully connected layer.

### 3.2 Train, test, and validation datasets

The training/testing dataset is generated from spectral libraries obtained from online repositories, NIST and MassIVE. The spectral libraries are preprocessed to generate two sets of data, i.e., spectra and their corresponding peptides. We obtained ∼ 4.8 million spectra with known peptide sources containing ∼ .5 million spectra from modified peptides. Train/Test split of 0.8/0.2 is applied such that no peptides are overlapping among the two splits. A separate spectral library is held out for validating the trained network, which is never used for training or testing purposes. Details of libraries used for train, test, and validation are provided in S1 File. Nine different types of modifications found in the training dataset are used for training the network. These modifications and their corresponding character values are given in Table 1. Details of the training dataset are given in Table 2.

**Table 1:** Modifications and the character values used in the training data. Nine modifications are used for training the network which are encoded with their corresponding character values to construct and modified peptide string.

Modifications	Values
Phospho	p
Oxidation	o
Deamidation	h
Carbamidomethyl	c
Acetyl	a
Ammonia-loss	r
Carbamyl	y
Dehydrated	d
Delta:H(2)C(2)	t

**Table 2:** Characteristics of training dataset used. Data was acquired from online repositories including NIST peptide library, MassIVE. The post translation modification in the dataset are CAM, Phosphorylation, Oxidation, and N-terminal Acetylation.

Parameters	Values
Training Samples	4.8M
Charge 2	2.6M
Charge 3	1.6M
Charge 4	0.4M
Other Charges	1.2M
Unmodified Samples	4.3M
Modified Samples	0.5M
Max Charge	8
Number of Species	7

Spectra are preprocessed into dense vectors (of length 80, 000) containing intensity values normalized to zero mean and unit variance. Each index of spectra vector represents an m/z bin of width 0.1 Da while the values at each index are the corresponding normalized intensities, hence each vector can hold a spectrum of up to 8, 000Da mass. Note that, due to the preprocessing step, the fragment tolerance is fixed to 0.1 Da for SpeCollate when performing database search. Peptide strings are padded with zero characters to the length of 64 before feeding to the PSN. The training/testing set is further split into batches of 1024 samples each. The training is performed using the PyTorch framework 1.6 running on python 3.7.4. For fast training, we perform the training process on NVIDIA V100s (32 GB SMX2) GPUs with 32GB of memory provided by Expanse SDSC supercomputer.

### 3.3 Training

The training process begins by a forward pass of a batch (a subset of 1024 data points) containing experimental spectra and their corresponding (positive) peptides through SSN and PSN, respectively. At this point, the dataset does not consist of sextuplets as the negatives examples have not been selected yet. Once a batch is forward passed through the network, the four negative examples for each positive pair (*q*_*i*_ ∈ *Q*, *p*_*i*_ ∈ *P*) are mined where *Q* is the set of embedded spectra, and *P* is the set of embedded peptides. A negative tuple (*q*_*j*_, *p*_*k*_) for *q*_*i*_ is mined such that *q*_*j*_ is the closest negative spectrum to *q*_*i*_ and *p*_*k*_ is the closest negative peptide to *q*_*i*_. Similarly, a negative tuple (*q*_*l*_, *p*_*m*_) for *p*_*i*_ is mined such that *q*_*l*_ is the closest negative spectrum to *p*_*i*_ and *p*_*m*_ is the closest negative peptide to *p*_*i*_. Hence, a sextuplet $S=(\left(q_{i},p_{i}\right),q_{j_{i}},p_{k_{i}},q_{l_{i}},p_{m_{i}})$ containing a query (or anchor) spectrum, positive peptide, two negative spectra and two negative peptides is constructed via online sextuplet mining for each positive example in the training dataset. The learning parameters are given in Table 3.

**Table 3:** Training parameters for SpeCollate.

Parameters	Values
Train/Test	0.8
Learning Rate	0.0001
Optimizer	Adam
Weight Decay	0.0001
Num. Layers	1 LSTM, 2 FC
Margin	0.2

#### 3.3.1 Online hardest sextuplet mining

Here we will derive the mathematical formulation of online negative mining to generate sextuplets. Given a batch *B* containing *b* training samples i.e. two sets $\bar{Q}$ and $\bar{P}$. After forwarding $\bar{Q}$ through SSN and $\bar{P}$ through PSN we get embedded spectra $Q=f_{SSN}\left(\bar{Q}\right)$ and peptides $P=f_{PSN}\left(\bar{P}\right)$ where $Q,P\subset {I\;R}^{256}$. To efficiently compute negative examples for each positive pair (*q*_*i*_ ∈ *Q*, *p*_*i*_ ∈ *P*), three distance matrices, *D*_*Q*×*Q*_, *D*_*Q*×*P*_, and *D*_*P*×*P*_, containing pairwise squared L2 distances (SED) of spectra and peptides are calculated. *D*_*Q*×*Q*_ contains the SED values between all spectra ‖*q*_*i*_−*q*_*j*_‖^2^, *D*_*Q*×*P*_ contains the SED values between spectra and peptides ‖*q*_*i*_ − *p*_*j*_‖^2^, and *D*_*P*×*P*_ contains SED values between all peptides ‖*p*_*i*_−*p*_*j*_‖^2^ where *i*, *j* ∈ {1, 2, …, *b*}. Note that these are symmetric matrices of size *b*×*b* with diagonal containing the positive pair SEDs for *D*_*Q*×*P*_ and zero for *D*_*Q*×*Q*_ and *D*_*P*×*P*_. For *D*_*Q*×*Q*_ and *D*_*P*×*P*_, we can calculate the distance matrix as follows (We will only show the calculation for *D*_*Q*×*Q*_ as *D*_*P*×*P*_ can be derived in exactly the same way:

Consider the Gramian matrix of *Q* is *G*_*Q*_:

$$\begin{array}{c} G_{Q}=Gramian\left(Q\right)=\left[\left\langle q_{i},q_{j}\right\rangle \right] \end{array}$$

and the diagonal of *G*_*Q*_ as:

$$\begin{array}{c} g_{Q}=diag\left(G_{Q}\right) \end{array}$$

Then *D*_*Q*×*Q*_ is given by:

$$\begin{array}{c} D_{Q\times Q}=g_{Q}1^{T}-2G_{Q}+1g_{Q}^{T} \end{array}$$

where **1** is a vector containing all ones and is the same length as *g*_*Q*_ i.e. *b*. *D*_*Q*×*P*_ can be derived in a similar fashion as follows:

Let

$$\begin{array}{c} G_{P}=Gramian\left(P\right)=\left[\left\langle p_{i},p_{j}\right\rangle \right] \end{array}$$

and

$$\begin{array}{c} g_{P}=diag\left(G_{P}\right) \end{array}$$

Then

$$\begin{array}{c} D_{Q\times P}=g_{Q}1^{T}-2Q^{T}P+1g_{P}^{T} \end{array}$$

Once these matrices are calculated, the four negatives can be obtained using the min function over matrices. Let the elements of matrices *D*_*Q*×*Q*_, *D*_*Q*×*P*_, and *D*_*P*×*P*_ be represented by *qq*_*ir*_, *pp*_*ir*_, and *qp*_*ir*_ respectively where *i*, *r* represent the row and the column indexes. Then the subscripts *j*_*i*_, *k*_*i*_, *l*_*i*_, and *m*_*i*_ for the negative examples in the sextuplet *S* can be determined using the following four equations:

$$\begin{array}{c} j_{i}=\underset{r,r\neq i}{\text{arg}\;\text{min}}\;q_{ir},\;i=1,\ldots ,b \end{array}$$

$$\begin{array}{c} k_{i}=\underset{r,r\neq i}{\text{arg}\;\text{min}}\;qp_{ri},\;i=1,\ldots ,b \end{array}$$

$$\begin{array}{c} l_{i}=\underset{r,r\neq i}{\text{arg}\;\text{min}}\;qp_{ir},\;i=1,\ldots ,b \end{array}$$

$$\begin{array}{c} m_{i}=\underset{r,r\neq i}{\text{arg}\;\text{min}}\;p_{ir},\;i=1,\ldots ,b \end{array}$$

**Fig 5:**
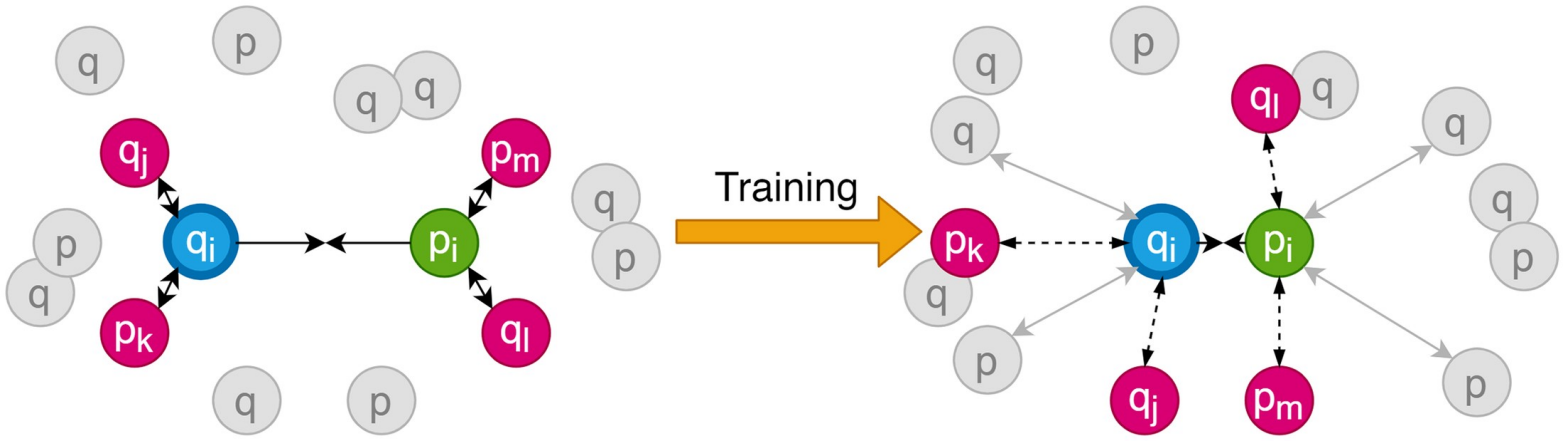
Online sextuplet mining for SNAP-loss. At each batch iteration, four negatives are selected that are closest to either q or p. The gradient update moves the negatives far away, and a new set of negatives is selected during the next iteration and so on. This process makes sure the network learns on the hardest examples for optimum training.

These subscripts indicate the corresponding indices of the negative spectra and peptides in sets *Q* and *P*; they can be directly accessed for loss calculation. Once all the sextuplets are constructed in a given batch, the loss value is computed using the custom-designed SNAP-loss function. The gradient update is back-propagated through both SSN and PSN. The online sextuplet mining is visualized in Fig 5.

### 3.4 SNAP-loss

The training objective is to minimize the SED between a given spectrum and its corresponding positive peptide while maximizing it for negative examples. To achieve this, we adopt a similar approach to the Triplet-Loss function [33] which works on triplets (A, P, N) with A as the anchor, P as the positive example and N as the negative example. In this case, the differences between SEDs among A and P ‖*A* − *P*‖^2^, and A and N ‖*A* − *N*‖^2^ is minimized with a constant margin value, added to the positive distance as shown below.

$$\begin{array}{c} L=\frac{1}{b}\sum_{i=0}^{b}\text{max}\left({\left\|A-P\right\|}^{2}-{\left\|A-N)\right\|}^{2}+margin,0\right) \end{array}$$

This approach works well where data with a single modality is dealt with, e.g., image verification in the case of FaceNet [29].

We design SNAP-loss which extends Triplet-Loss to multi-modal data, in our case numerical spectra and sequence peptides. For this purpose, we consider all possible negatives (*q*_*j*_, *p*_*k*_, *q*_*l*_, *p*_*m*_) for a given positive pair (*q*_*i*_, *p*_*i*_) and average the total loss. The four possible negatives are explained below:

- *q*_*j*_: The negative spectrum for *q*_*i*_.
- *p*_*k*_: The negative peptide for *q*_*i*_.
- *q*_*l*_: The negative spectrum for *p*_*i*_.
- *p*_*m*_: The negative peptide for *p*_*i*_.

To calculate the loss value, we first define a few variables that are precomputed in distances matrices above as follows:

Let

$$\begin{array}{c} d_{i}={\left\|q_{i}-p_{i}\right\|}^{2} \end{array}$$

$$\begin{array}{c} d_{n1}={\left\|q_{i}-q_{j}\right\|}^{2} \end{array}$$

$$\begin{array}{c} d_{n2}={\left\|q_{i}-p_{k}\right\|}^{2} \end{array}$$

$$\begin{array}{c} d_{n3}={\left\|p_{i}-q_{l}\right\|}^{2} \end{array}$$

$$\begin{array}{c} d_{n4}={\left\|p_{i}-p_{m}\right\|}^{2} \end{array}$$

Then the SNAP-loss is calculated for a batch of size *b* as follows:

$$\begin{array}{c} L=\frac{1}{4b}\sum_{i=1}^{b}\sum_{r=1}^{4}\text{max}\left(d_{i}-d_{nr}+margin,0\right) \end{array}$$

The training process is visualized in Fig 5.

### 3.5 Similarity inference

Once the training is complete, we perform the similarity inference on the test dataset by simply transforming the peptides and spectra into the embedded subspace and applying the nearest neighbor search. Fig 3 shows the resultant euclidean space is $R^{256}$, where all the peptides and spectra are projected onto.

#### 3.5.1 Indexing

Since a large number of spectra might need to be searched against peptides, we can index the peptides by precomputing the embedded feature vectors and store them for later use. Similar pre-computation can be performed for the experimental spectra before performing the search to avoid repeated encoding, as each experimental spectrum needs to be searched against multiple peptides.

The L2 distance measure can be efficiently calculated on the GPU by computing the masked distance matrix for the peptides that fall within the precursor m/z range. Furthermore, this process can easily scale to multiple GPUs, making it feasible for large datasets. The inverse of the L2 distance (1/*L*2) is reported as the match score.

#### 3.5.2 L2 distance measure

To measure the L2 distance between the embedded set of spectra *Q* and peptides *P*, we split *Q* into batches of size 1024. On the other hand, peptides are selected for each batch of spectra based on the precursor tolerance, and their number can vary. We limit the maximum number of peptides in a batch to 16384 due to the memory limit (32 GBs) of the GPU. If more than 16384 peptides fall within the precursor window, they are further split into sub-batches, and the search process is repeated for each sub-batch. As a result, we have two matrices *A*_1024×256_ and *B*_<16384×256_ containing a batch of spectra and a sub-batch of peptides, respectively. Parallel distance matrix *D*_*A*×*B*_ calculation is performed using the following equation:

$$\begin{array}{c} D_{A\times B}=g_{A}1^{T}-2A^{T}B+1g_{B}^{T} \end{array}$$

where *g*_*A*_ is the diagonal vector of the Gramian matrix *G*_*A*_ of *A* and *g*_*B*_ is the diagonal vector of the Gramian matrix *G*_*B*_ of *B*. *D*_*A*×*B*_ is a 1024× ≤ 16384 distance matrix and contains the distances of each spectrum in *A* to each peptide in *B*, i.e., each row in *D*_*A*×*B*_ represents an experimental spectrum while each column represents a peptide. Since not all peptides lie within the precursor window of every spectrum, we compute a mask matrix *M* of the same size as *D*_*A*×*B*_ which contains 1 for peptides that fall within the precursor window of each spectrum and 0 for the rest. Matrix *M* is calculated dynamically based on the user-provided precursor tolerance (Da or ppm). Hadamard product of *D*_*A*×*B*_ and *M* gives the distance measure of only relevant peptide-spectrum pairs. For each spectrum, 5 top-scoring peptide (minimum distance) are kept, and the rest are discarded, giving a resultant score matrix of size 1024×5, which is stored for posterior analysis later.

## 4 Experiments & results

In this section, we will discuss the experimental setup, present results, and provide a discussion.

### 4.1 Experimental setup

We train our network for 200 epochs on a dataset of size ∼ 4.8 million sextuplets. The training is performed on an NVIDIA V100s (32 GB SMX2) GPUs with 32GB of memory provided by Expanse SDSC supercomputer installed in nodes with up to 4 GPU nodes, 40 CPU cores, and 384 GBs of memory (10 CPU cores and 96 GBs per GPU node). We use PyTorch 1.6 to design our network using python 3.7.4 on Linux 16.04. We also perform the database search on the test dataset to measure the quality of results just by comparing the embeddings.

### 4.2 Training and model evaluation

**Fig 6:**
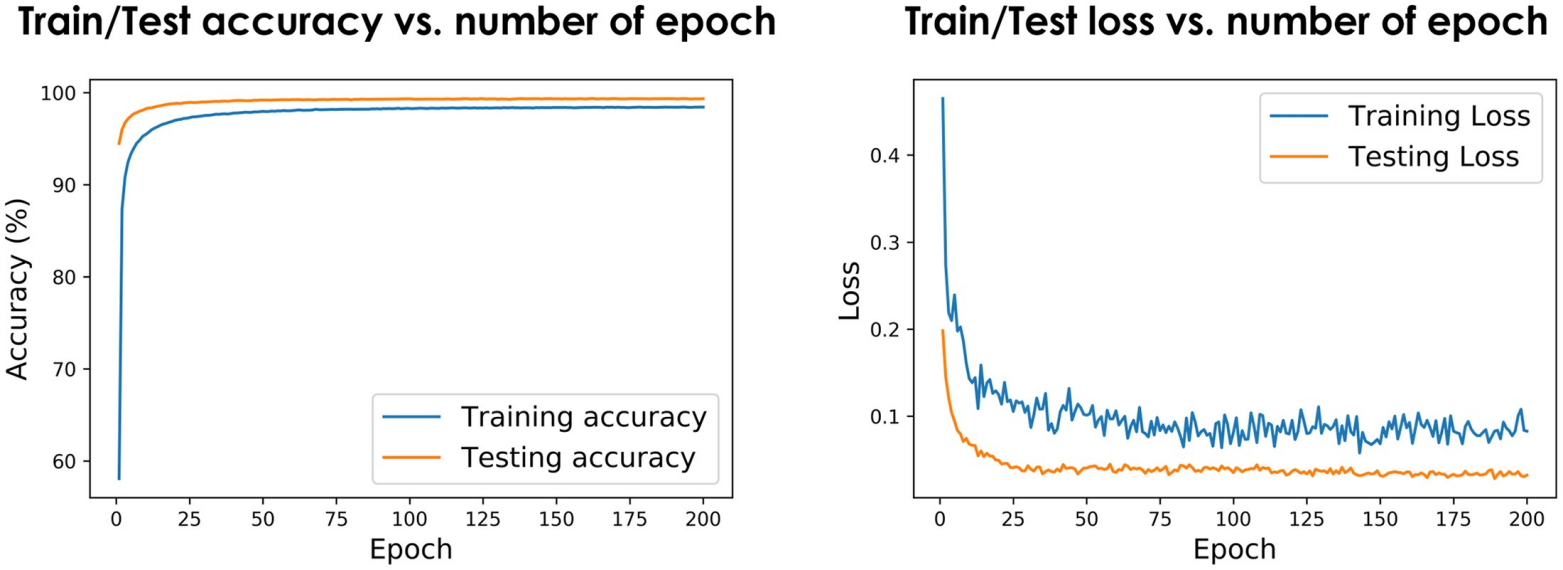
Training progress for train/test data (left) and the loss value over time (right).

We train the network for 200 epochs to achieve validation accuracy of 95.5%. The accuracy is measured by the ratio of the number of times the correct peptide is the closest one to the anchor spectrum to the total number of spectra in a batch. Let us define the true peptide *tp* as a Boolean function which outputs 1 if the closest peptide *p* to the anchor *q* in the current batch *B* is the true peptide *p*_*q*_ and zero otherwise.

$$\begin{array}{c} tp\left(q,B\right)=\{\begin{array}{cc} 1 & {\text{arg}\;\text{min}}_{p\in B}\;{\left\|q-p\right\|}^{2}=p_{q}\\ 0 & \text{otherwise} \end{array} \end{array}$$

$$\begin{array}{c} Accuracy=\frac{\sum_{q\in B}tp\left(q,B\right)}{\left|B\right|} \end{array}$$

where *p*_*q*_ is the true peptide for *q*, *B* is the current batch, and *P*_*B*_ and *Q*_*B*_ represent the peptides and spectra in *B* respectively. Fig 6 shows the training process in terms of accuracy of training and validation data as well the loss value.

**Fig 7:**
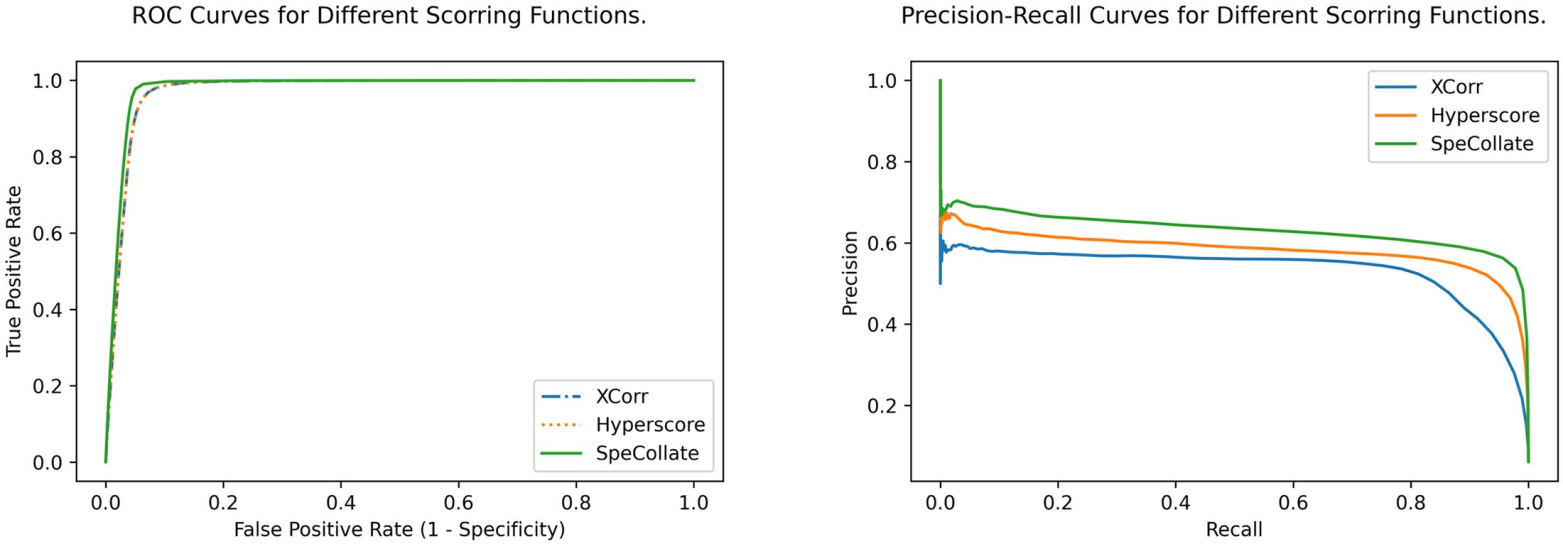
ROC (left) and Precision-Recall (right) curves for closed search. Both ROC curves and Precision-Recall curves for SpeCollate, XCorr, and Hyperscore show that SpeCollate performs best for all cutoff values for closed search (±0.5 Da precursor mass tolerance).

We plot the ROC curves as well as Precision-Recall curves for comparing the performance of the three scoring functions. As shown in Fig 7, SpeCollate performs significantly better than XCorr and Hyperscore in closed search. Note that ROC curves tend to overestimate the model’s skill when the classes are not balanced, and there are far more true negatives than false positives. Therefore, for a scenario where positive examples are far more valuable than the negative ones (such as when searching for a peptide-spectrum match), Precision-Recall curves better represent the performance as the true-negatives are not considered for either precision or recall calculation.

**Fig 8:**
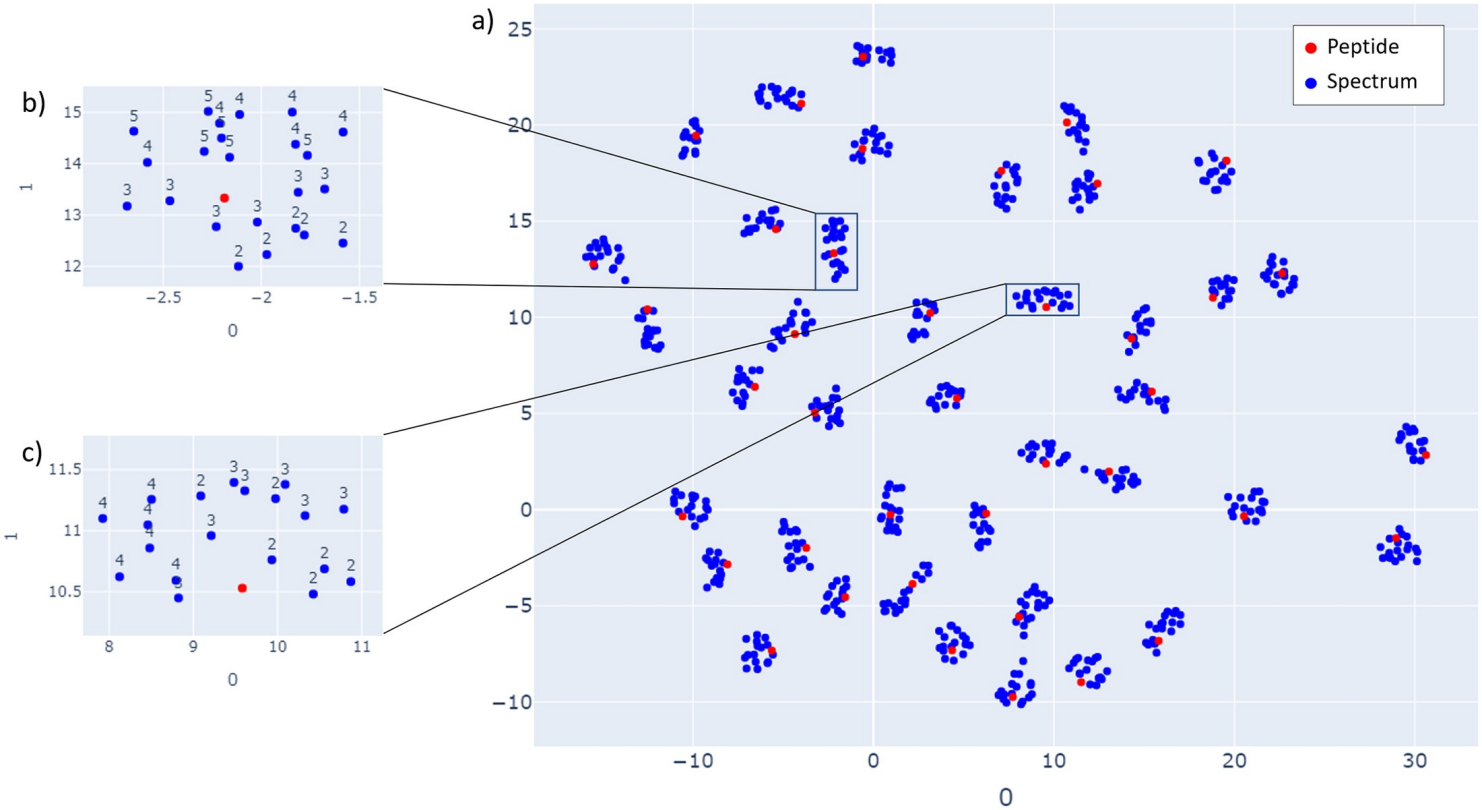
2D UMAP visualization of embedded spectra and peptide vectors generated by SpeCollate. a) Clustering of spectra with different precursor charges around their corresponding peptides as well as the separation of clusters within a mass-range is shown for 40 unique peptides. The red point represents the peptide within a cluster, while the blue points represent spectra (with different charge values) corresponding to the peptide. Sub-figures b) and c) show the close-up of two clusters with charge labeling of spectra. As can be seen, spectra with all charges are mapped close to the peptide. However, spectra with higher precursor charge values are mapped relatively farther than those with smaller values.

Fig 8, provides the UMAP [34] visualization of spectra and peptide embeddings. Embeddings are generated for library spectra provided by Proteome Tools by first sorting the spectra according to their precursor mass and then selecting spectra for 40 unique peptides, sequentially, for precursor mass value of ≥ 2000 Da., such that the number of spectra for each peptide is ≥ 15 to understand how SpeCollate maps spectra of different charges (2–5) close to the corresponding peptide embeddings. Selecting spectra that are next to each other in precursor mass helps us visualize the separation of different clusters within a precursor mass window when performing the database search. Similar visualizations for different precursor mass values (1500–3000) are provided in S3 File.

### 4.3 Database search

The model is also tested in a complete proteomics pipeline using the dataset from NIST as well as three real-world datasets (PXD000612 [35], PXD009861 [36], PXD001468 [37]), obtained from PRIDE, by performing database search against the human proteome and obtaining PSMs and peptides reported under 1% FDR. The performance is compared against Crux [38] and MSFragger [7] by performing separate target decoy database searches and FDR estimation using Percolator [39]. Closed search is performed with precursor mass tolerance of ±5ppm. Note that fragment tolerance is fixed to 0.1 as MS/MS spectra are preprocessed into arrays with each index representing a bin width of 0.1 Da. The protein database is in-silico digested using tryptic digestion, allowing up to two missed cleavages and 7–50 amino acids per peptide. Different peptide databases with zero and up to one and two phosphorylation and oxidation sites per peptide are generated and searched against different datasets. The decoy entries are generated by reversing the target peptides (except the first and the last amino acid) and removing any overlapping peptides from the decoy database. The resulting peptide databases contain ∼ 2.7M, ∼ 11.7M, and ∼ 32.3M peptides for zero, one and two phosphorylation sites respectively while for oxidation, the peptide databases contain ∼ 2.7M, ∼ 3.9M, and ∼ 4.2M peptides. The search is performed by generating theoretical spectra of fixed intensity values and reporting 5 top-scoring peptide spectrum matches per spectrum for each target and decoy database. Percolator/PeptideProphet then reorders these PSMs to estimate false-discovery rate, and a subset of PSMs and peptides estimated to have FDR < 1% is reported. As SpeCollate can only generate charge-independent peptide embeddings, we limit the fragment charge of theoretical spectra generated by Crux and MSFragger to +1 for a fair comparison.

#### 4.3.1 Search results and comparisons

**Fig 9:**
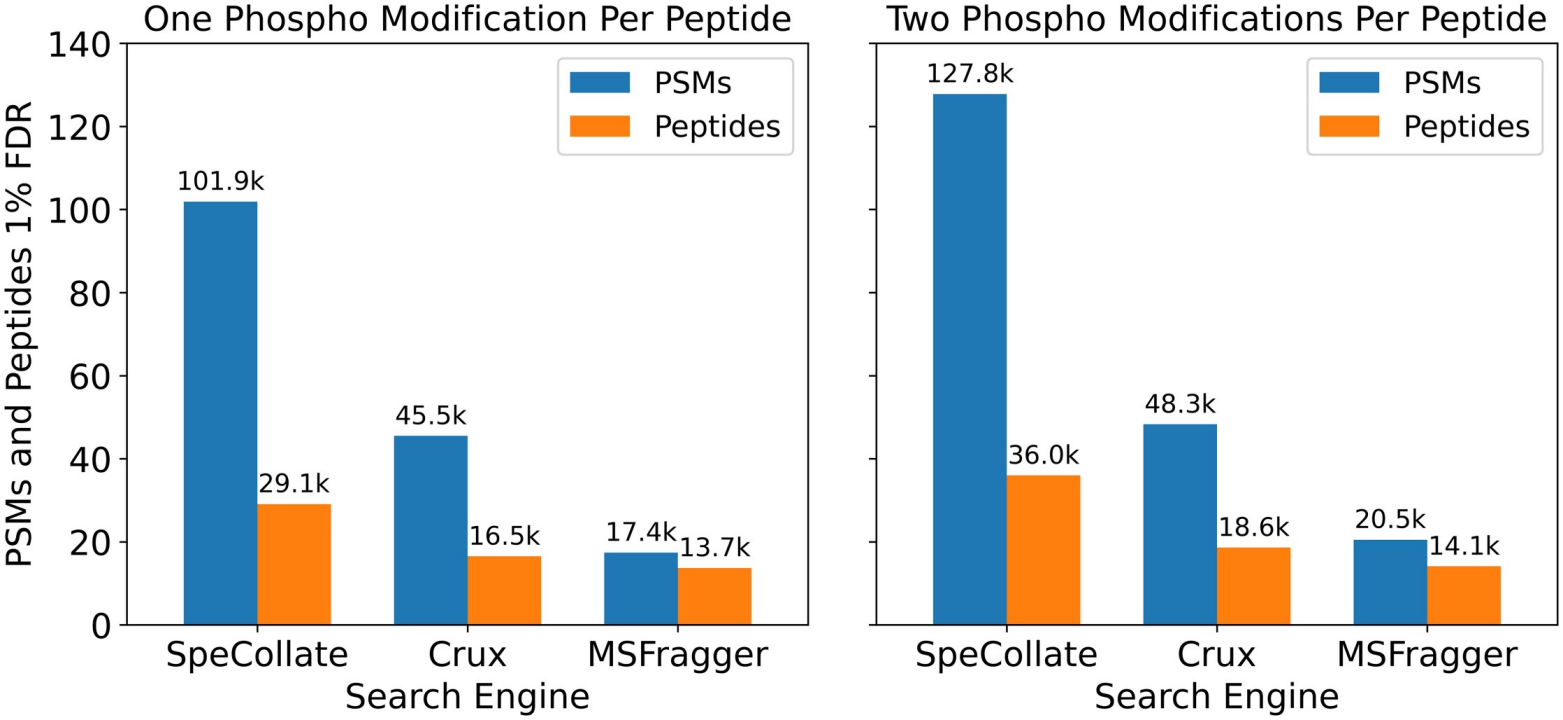
Closed search (±5ppm) comparison against Crux and MSFragger using NIST Dataset with one phosphorylation modification per peptide (left) and two phosphorylation modifications (right). SpeCollate significantly outperforms Crux and MSFragger in both PSMs and number of peptides.

Fig 9 provides a comparison of SpeCollate’s performance against Crux and MSFragger for NIST “Human HCD Phospho” library spectra. For the left subplot, the peptides are generated with up to one phosphorylation modification per peptide, while in the right subplot, up to two phosphorylation modifications per peptide are allowed. SpeCollate outperforms both Crux and MSFragger in terms of the number of identified PSMs and peptides under 1% FDR. We do not perform a search with no modifications as the NIST dataset being searched contains only phosphorylated spectra.

**Fig 10:**
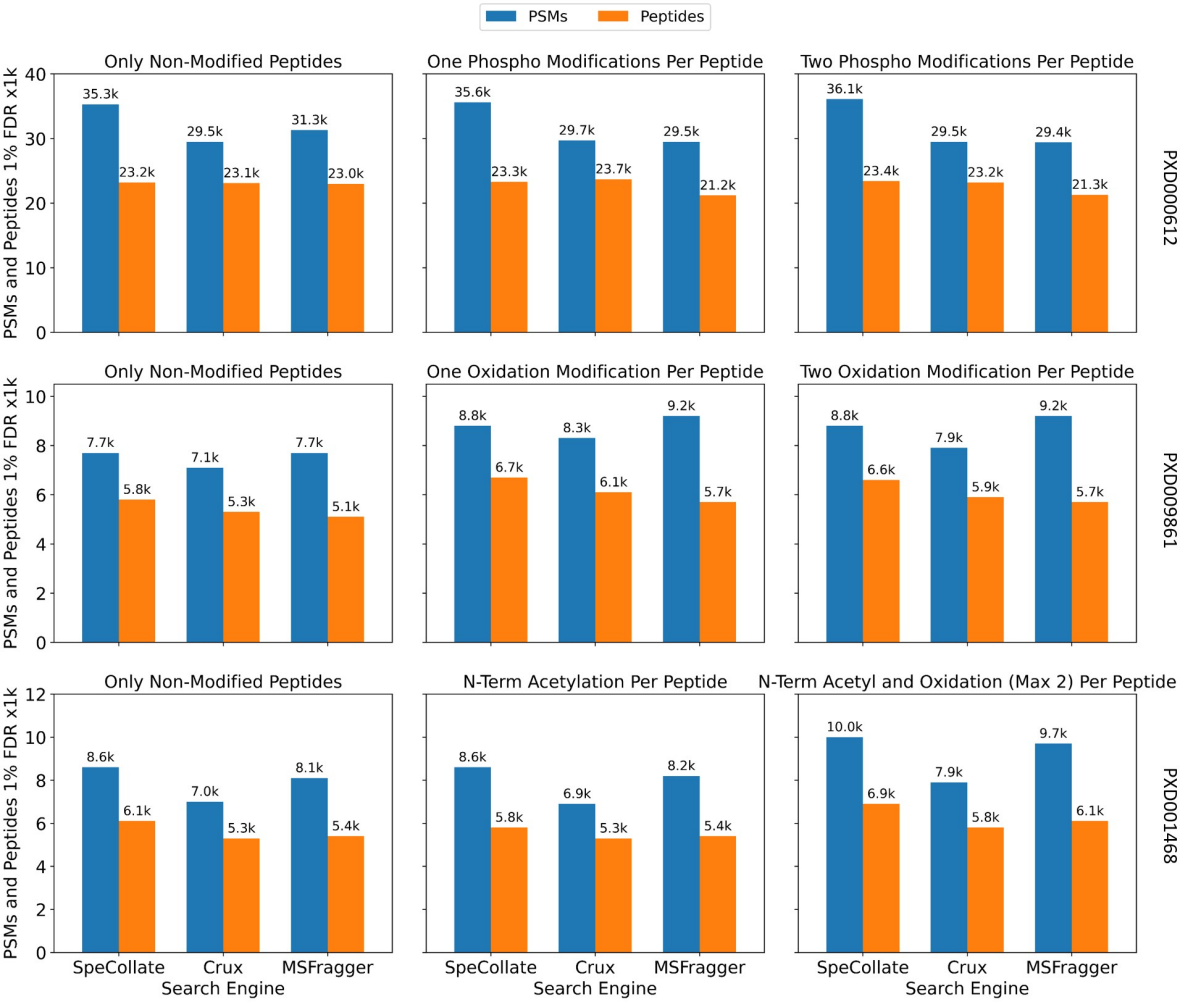
Closed search (±5ppm) comparison against Crux and MSFragger using real-world data from PRIDE repository PXD000612 (top), PXD009861 (middle), and PXD001468 (bottom). For PXD000612, the search is performed against a peptide database generated from the human proteome with zero and up to one and two phosphorylation (amino acids S, T, and Y) modifications for subplots left, middle, and right, respectively. For dataset PXD009861, the search is performed against databases with a different number of oxidized M-residues (up to two per peptide), while for dataset PXD001468, the search is performed against a database with one n-term acetylation and up to two oxidation sites (max two modifications per peptide). SpeCollate can outperform Crux and MSFragger in terms of PSMs while giving a comparable performance in terms of the number of peptides.

Next we perform the comparison using real-world datasets PXD000612 [35], PXD009861 [36], and PXD001468 [37] in Fig 10 (See S2 File for details about these datasets). For the PXD000612 dataset, the search is performed against different number of phosphorylation sites (up to two) added to the peptide database. In contrast, for dataset PXD009861, the search is performed against databases with different number of oxidized M-residues (up to two per peptide) while for dataset PXD001468, the search is performed against database with one n-term acetylation and up to two oxidation sites (max 2 modifications per peptide). SpeCollate is able to outperform Crux and MSFragger in all experiments except for PXD000612 searched against the single phosphorylated database where the number of peptides identified by SpeCollate is slightly less than Crux. Comparison using default XCorr and MSFragger settings (i.e., allowing theoretical fragments with > +1 charge) is provided in S5 File.

**Fig 11:**
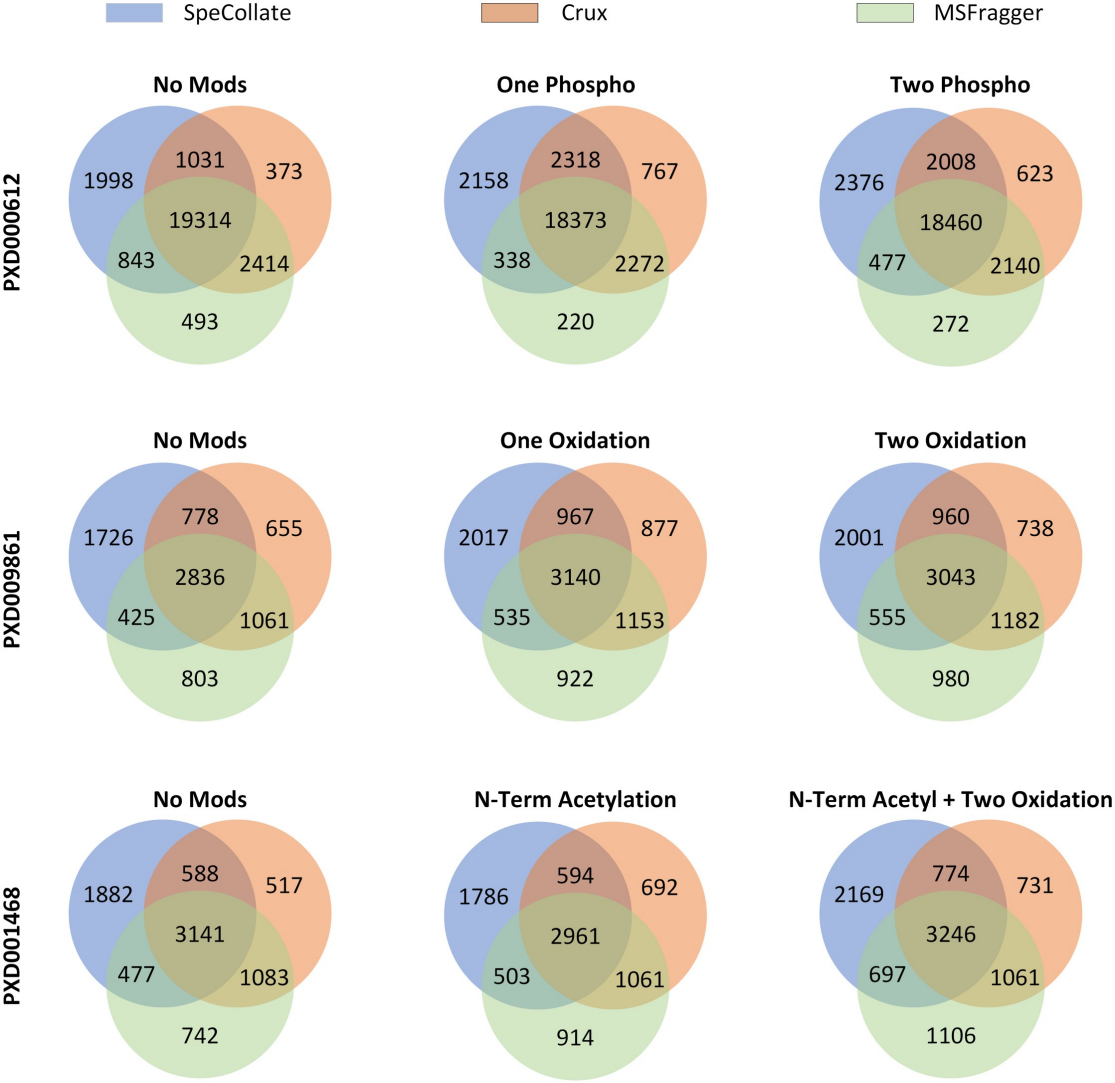
Venn diagrams showing the overlap of peptides among SpeCollate, Crux, and MSFragger. As can be seen, most of the peptides are common among the three tools while SpeCollate does identify most unique peptides compared to Crux and MSFragger. See S4 File for list of peptides identified in each experiment.

We also investigate the overlap of identified peptides by SpeCollate, Crux, and MSFragger using Venn diagrams as shown in Fig 11 for each experiment performed in Fig 10. As can be seen, most of the peptides for each experiment are common among the three search tools. However, SpeCollate does identify more unique peptides than Crux and MSFragger. Generally, we discovered that compared to Crux and MSFragger, unique peptides identified by SpeCollate were mostly of smaller length 10. On the other hand, peptides identified by Crux and MSFragger (and missed by SpeCollate) followed similar length distribution to the overall set of identified peptides. On the other hand, the precursor charge distribution of unique peptides for each tool remains similar as the identified peptides are mostly of lower charge. As expected, both XCorr and MSFragger identify greater number of unique peptides with higher precursor charge when their default settings are used (i.e. theoretical fragments with > +1 charge are allowed).

## 5 Discussion

SpeCollate provides a stepping stone towards mass-spectrometry-based proteomics database search using deep learning. In contrast to previous approaches, SpeCollate maps both the spectra and peptide into an embedding which can then directly be compared with each other and eliminates the need for scoring functions and spectrum simulators. Our proposed cross-model method is then shown to work with deep learning methods that can outperform the state-the-of-art techniques. Through extensive experimentation using spectral libraries and real-world MS/MS data, we demonstrate the efficiency of our cross-modal learning technique, presenting further opportunities to the research community for exploring simulator-less peptide database search implementations.

### 5.1 Limitations and future directions

Currently, SpeCollate is trained to match spectra to their exact peptide sequence, i.e., modified spectra are not embedded close to the non-modified peptide. Moreover, peptide embeddings are charge-independent, and the model only generates one embedding per peptide. Although spectra with a higher precursor charge > 2 are mapped close to the peptide, they tend to be relatively farther than precursor charge 2. One of the future goals of our work is to enable open-search using deep learning, which in addition to identifying modified spectra, can also be used as a pre-search filter to extract a subset of relevant peptides. Furthermore, our next step is to enable charge-specific peptide embeddings to map spectra with different charges to their corresponding peptides efficiently. To this end, several research problems remain open, including curating improved training datasets, improving the network architecture to better capture the input domain, employing adversarial learning for bridging the modality gap between peptides and spectra and improving the training process by redesigning the loss-function from the ground up.

## 6 Conclusion

This paper designs and implements SpeCollate, a deep similarity network for proteomics, to learn a cross-modal similarity function between peptides and spectra to identify peptide-spectrum matches. Proteomics has entered the realm of Big-Data, and the number of available labeled and annotated spectra is increasing rapidly, enabling sophisticated models to be trained. SpeCollate provides a data-oriented algorithm design for peptide database search, eliminating the inherent problems associated with numerical strategies. This is achieved by learning a cross-modal similarity function that embeds spectra and peptides in a shared euclidean subspace for direct comparison. SpeCollate learns the similarity function by optimizing a custom-designed loss function, SNAP-loss, which trains on sextuplets of data points to project positive examples closer to each other while pushing negative examples far from each other. By training on 4.8 million sextuplets, SpeCollate is able to achieve a remarkable test accuracy of 95.5%. Although the test accuracy is relatively high, there is still room for real-world database search accuracy improvement. This can be improved by fine-tuning BiLSTM and generating better training data to enable the network to learn more features in future work.

## Supporting information

S1 File Train test validation libraries. Details of libraries used in training, testing, and validating SpeCollate.
(XLSX)

S2 File Datasets. Datasets used to compare SpeCollate’s performance against Crux and MSFragger.
(XLSX)

S3 File UMAP. UMAP projections of embedded peptides and their corresponding spectra at different mass ranges.
(PPTX)

S4 File Peptides. List of peptides identified for different experiments.
(XLSX)

S5 File Comparison. Comparison of SpeCollate with Default XCorr and MSFragger Settings.
(DOCX)
