# Supplementary material for: SpeCollate: Deep cross-modal similarity network for mass spectrometry data based peptide deductions
Source: PLoS One. 2021 Oct 29;16(10):e0259349. doi: 10.1371/journal.pone.0259349 (PMC8555789; doi:10.1371/journal.pone.0259349)
Supplement: S3 File — UMAP projections of embedded peptides and their corresponding spectra at different mass ranges. (PPTX) [file pone.0259349.s003.pptx]

## Slide 1
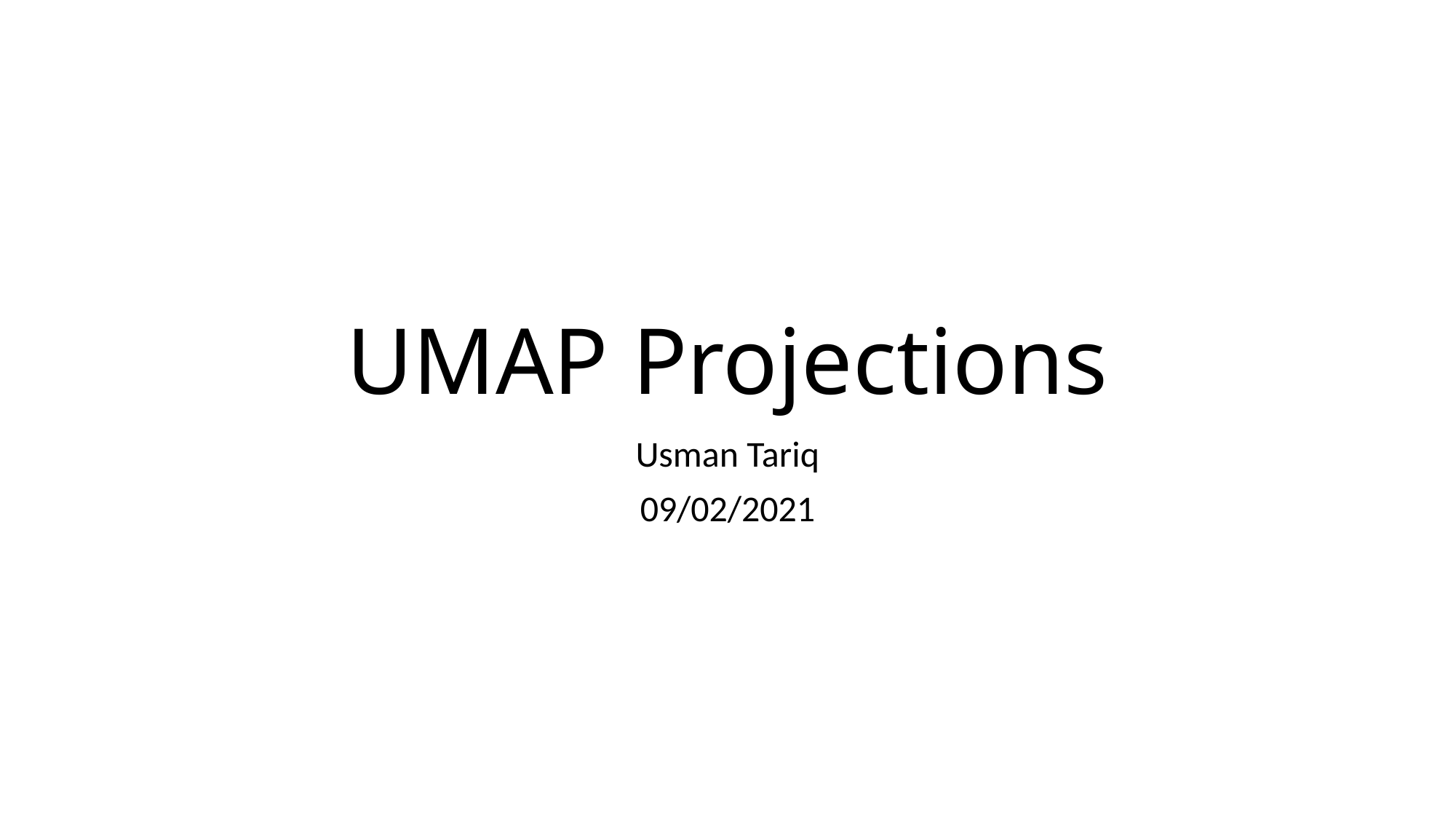

# UMAP Projections
Usman Tariq
09/02/2021

## Slide 2
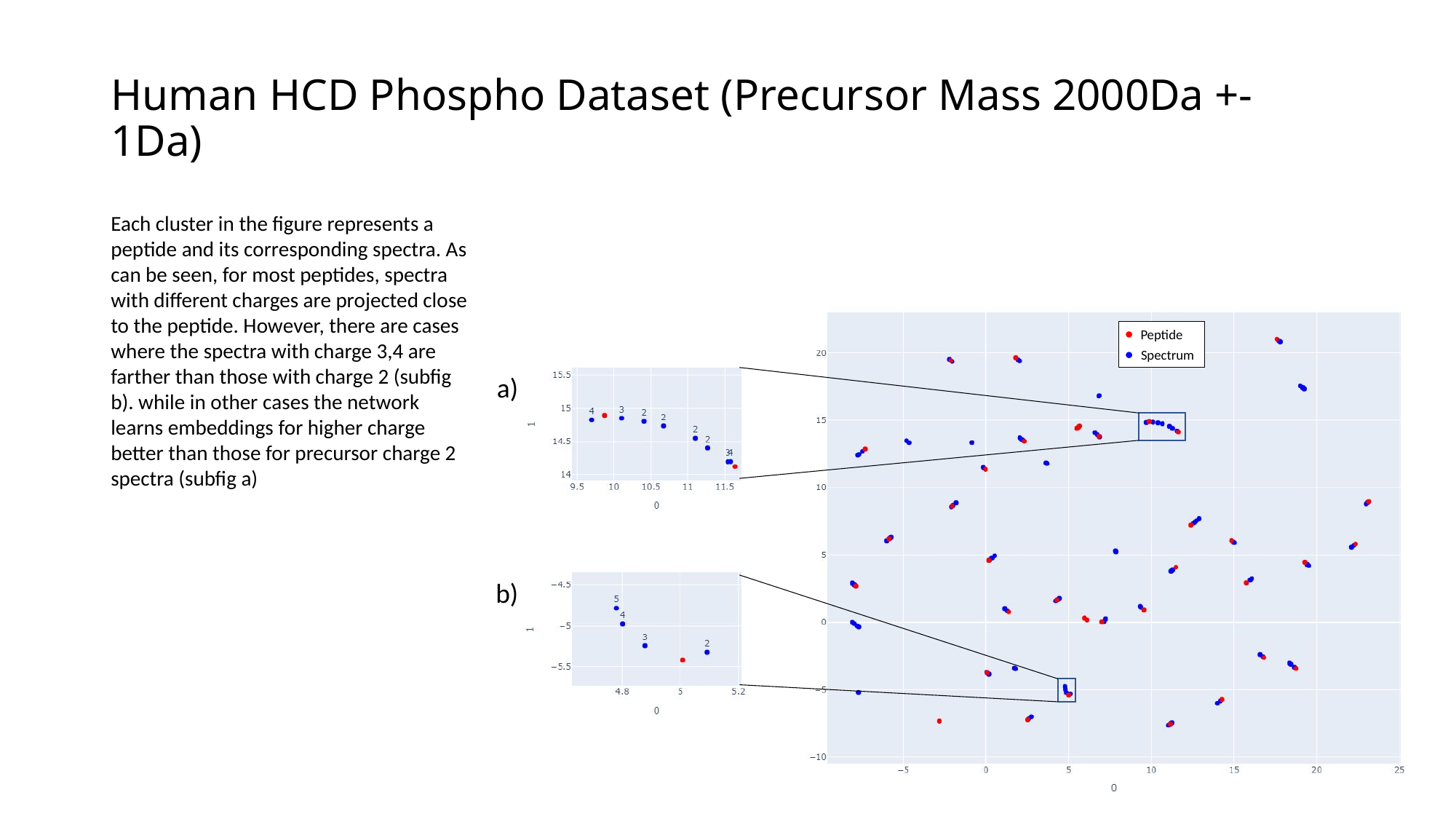

# Human HCD Phospho Dataset (Precursor Mass 2000Da +- 1Da)
Each cluster in the figure represents a peptide and its corresponding spectra. As can be seen, for most peptides, spectra with different charges are projected close to the peptide. However, there are cases where the spectra with charge 3,4 are farther than those with charge 2 (subfig b). while in other cases the network learns embeddings for higher charge better than those for precursor charge 2 spectra (subfig a)
Peptide
Spectrum
a)
b)

## Slide 3
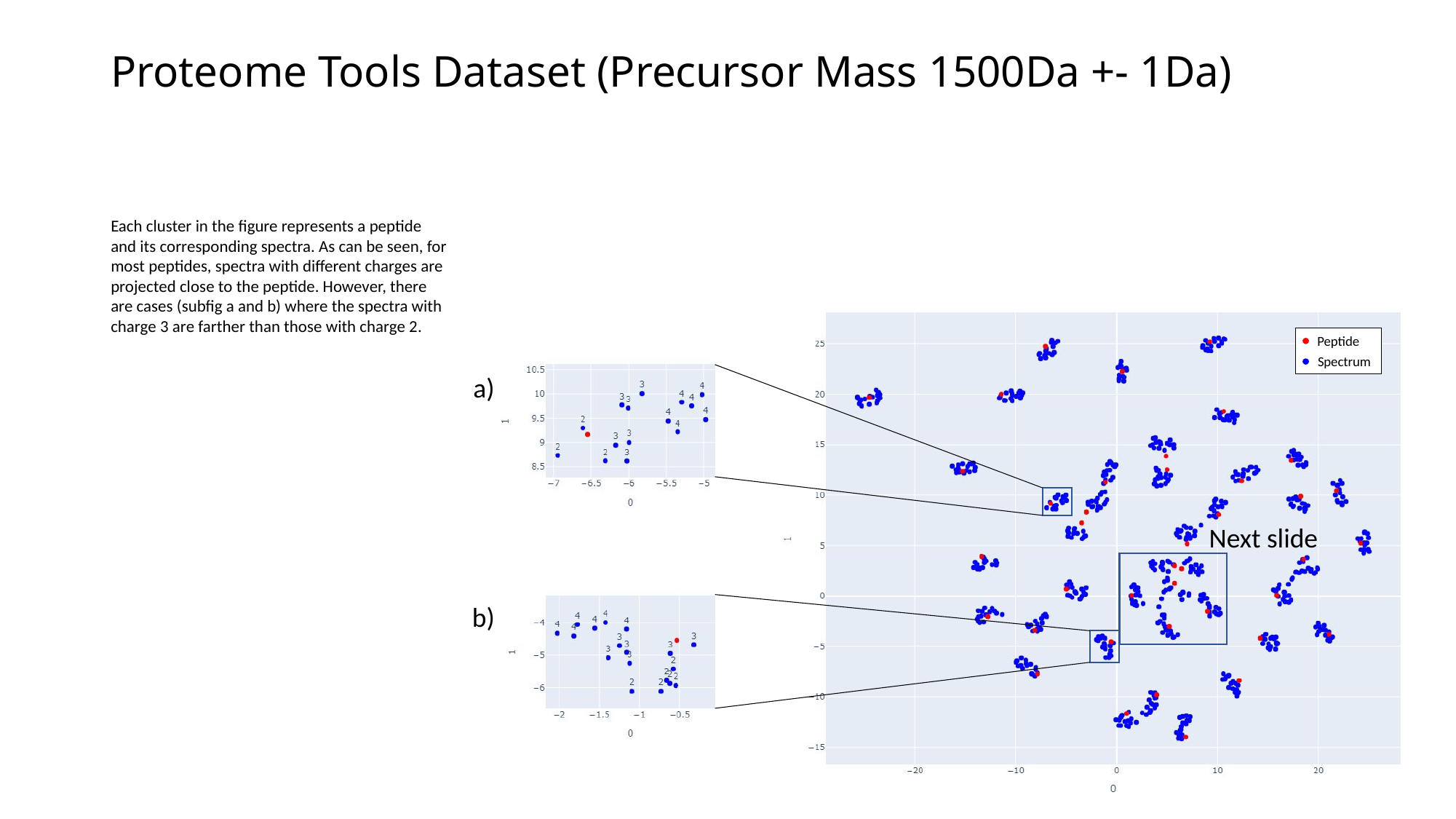

# Proteome Tools Dataset (Precursor Mass 1500Da +- 1Da)
Each cluster in the figure represents a peptide and its corresponding spectra. As can be seen, for most peptides, spectra with different charges are projected close to the peptide. However, there are cases (subfig a and b) where the spectra with charge 3 are farther than those with charge 2.
Peptide
Spectrum
a)
Next slide
b)

## Slide 4
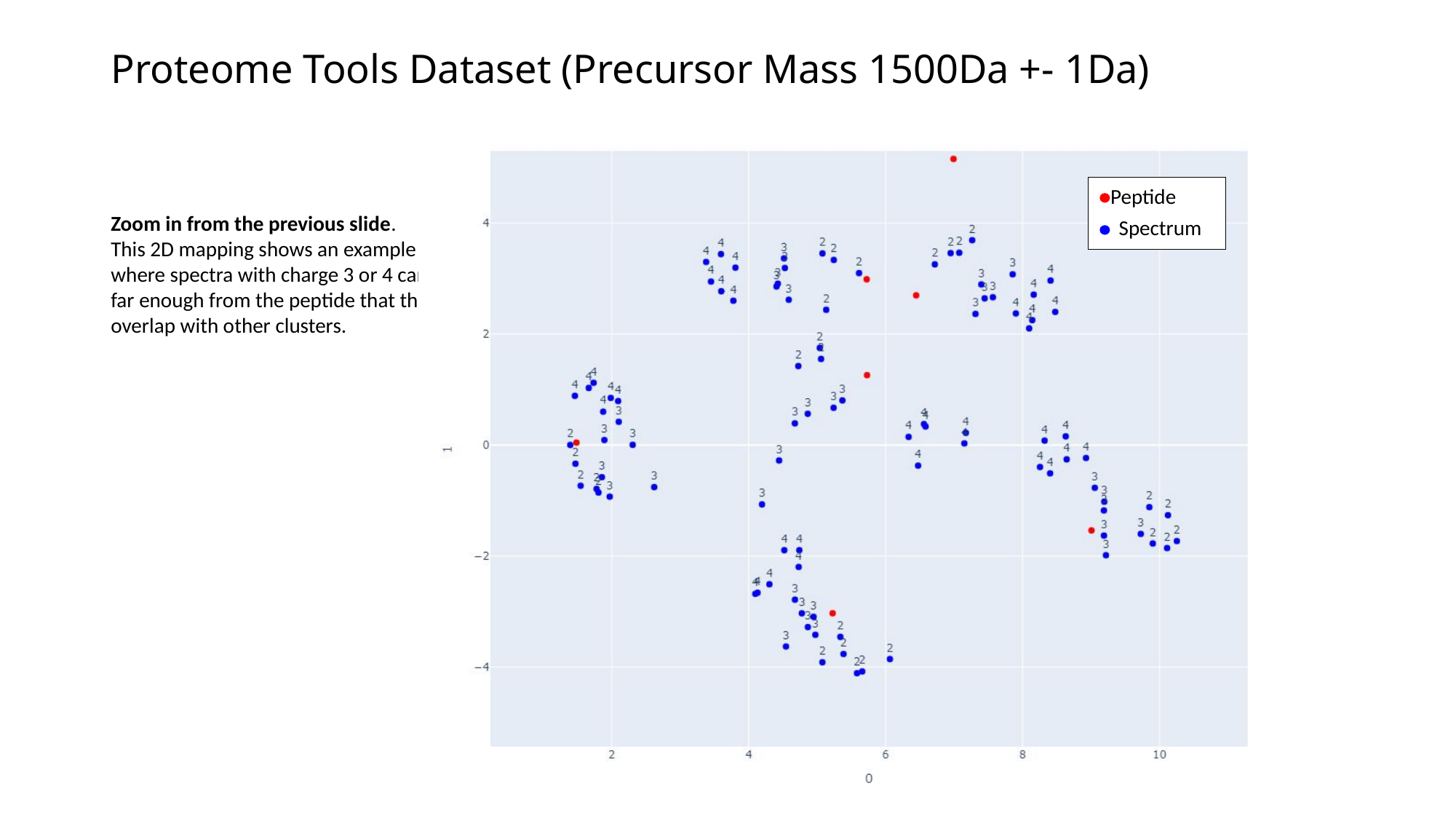

# Proteome Tools Dataset (Precursor Mass 1500Da +- 1Da)
Peptide
Spectrum
Zoom in from the previous slide.
This 2D mapping shows an example where spectra with charge 3 or 4 can map far enough from the peptide that they can overlap with other clusters.

## Slide 5
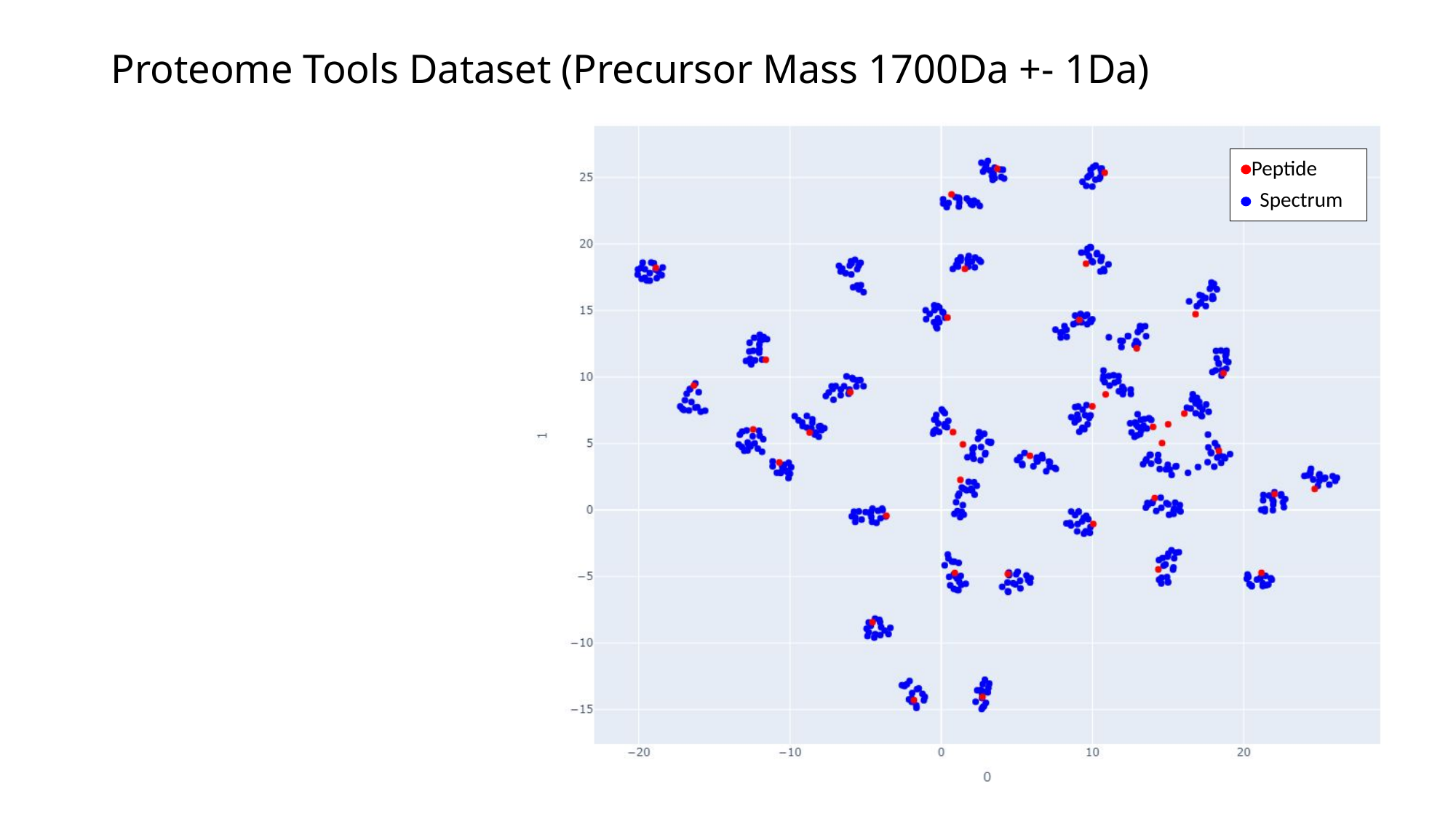

# Proteome Tools Dataset (Precursor Mass 1700Da +- 1Da)
Peptide
Spectrum

## Slide 6
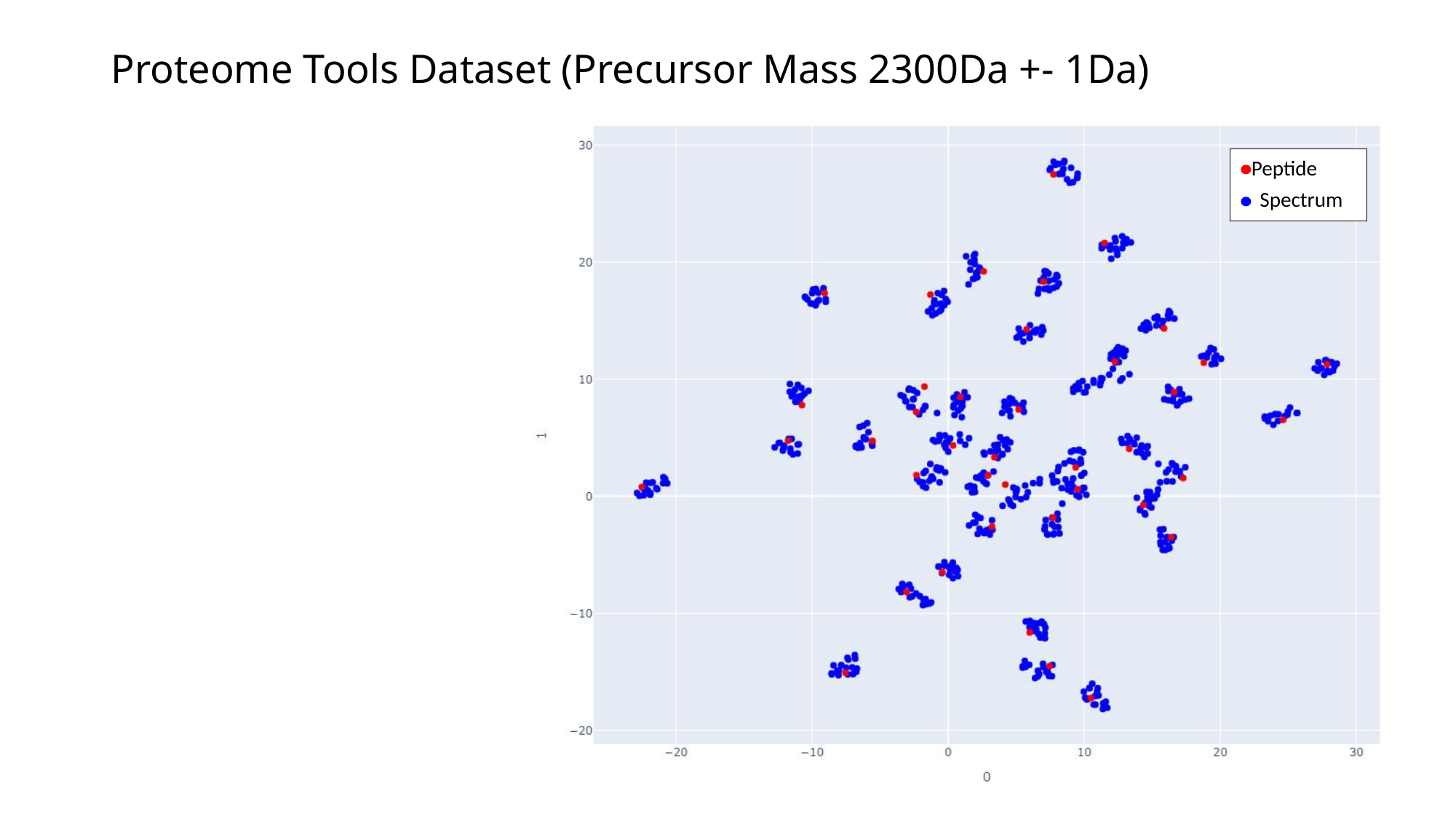

# Proteome Tools Dataset (Precursor Mass 2300Da +- 1Da)
Peptide
Spectrum

## Slide 7
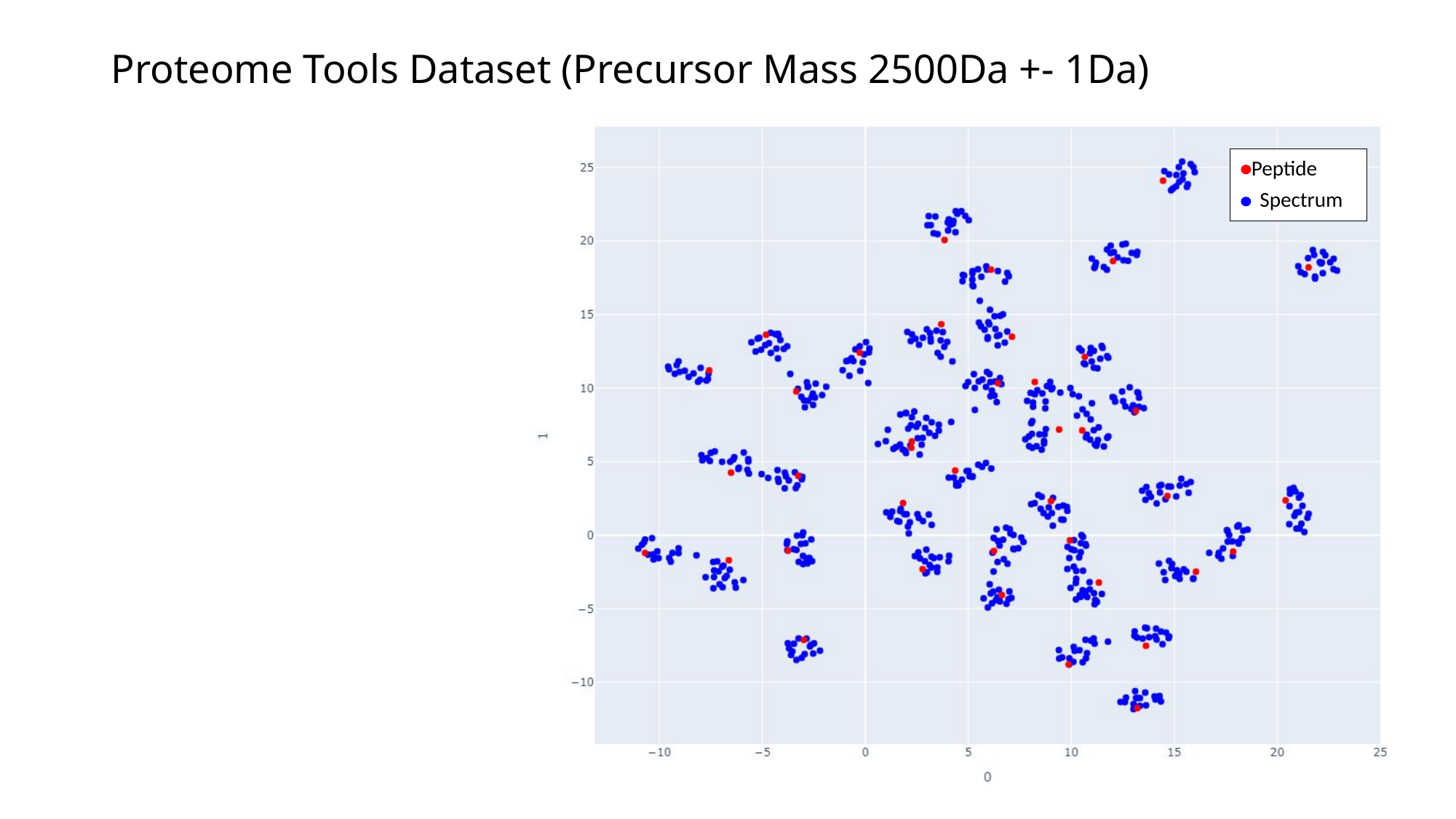

# Proteome Tools Dataset (Precursor Mass 2500Da +- 1Da)
Peptide
Spectrum
