# Supplementary material for: SpeCollate: Deep cross-modal similarity network for mass spectrometry data based peptide deductions
Source: PLoS One. 2021 Oct 29;16(10):e0259349. doi: 10.1371/journal.pone.0259349 (PMC8555789; doi:10.1371/journal.pone.0259349)
Supplement: S5 File — Comparison of SpeCollate with Default XCorr and MSFragger Settings. (DOCX) [file pone.0259349.s005.docx]

**Comparison of SpeCollate with XCorr and MSFragger using Default Settings**

**Comparison using NIST Human HCD Phosphorylated dataset:**


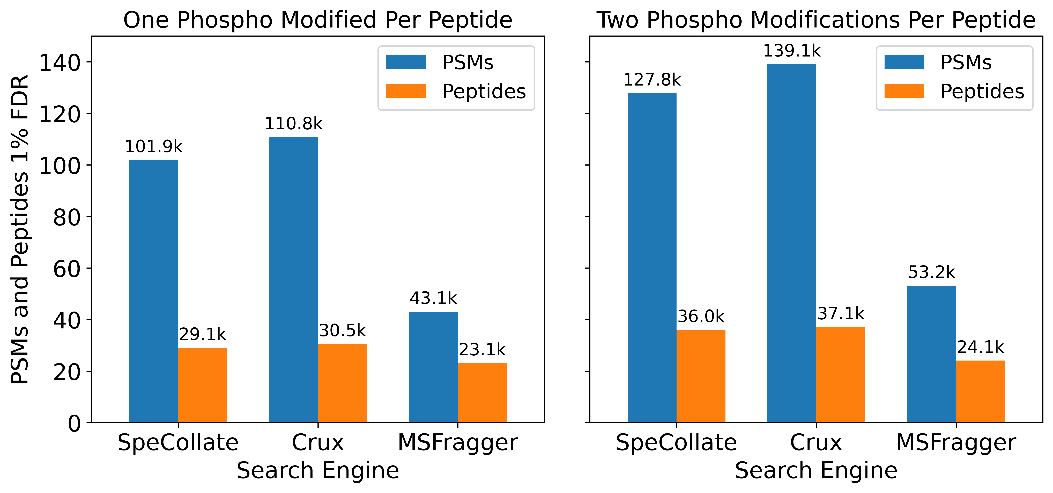


**Comparison using PXD000612, PXD009861, PXD001468 datasets:**


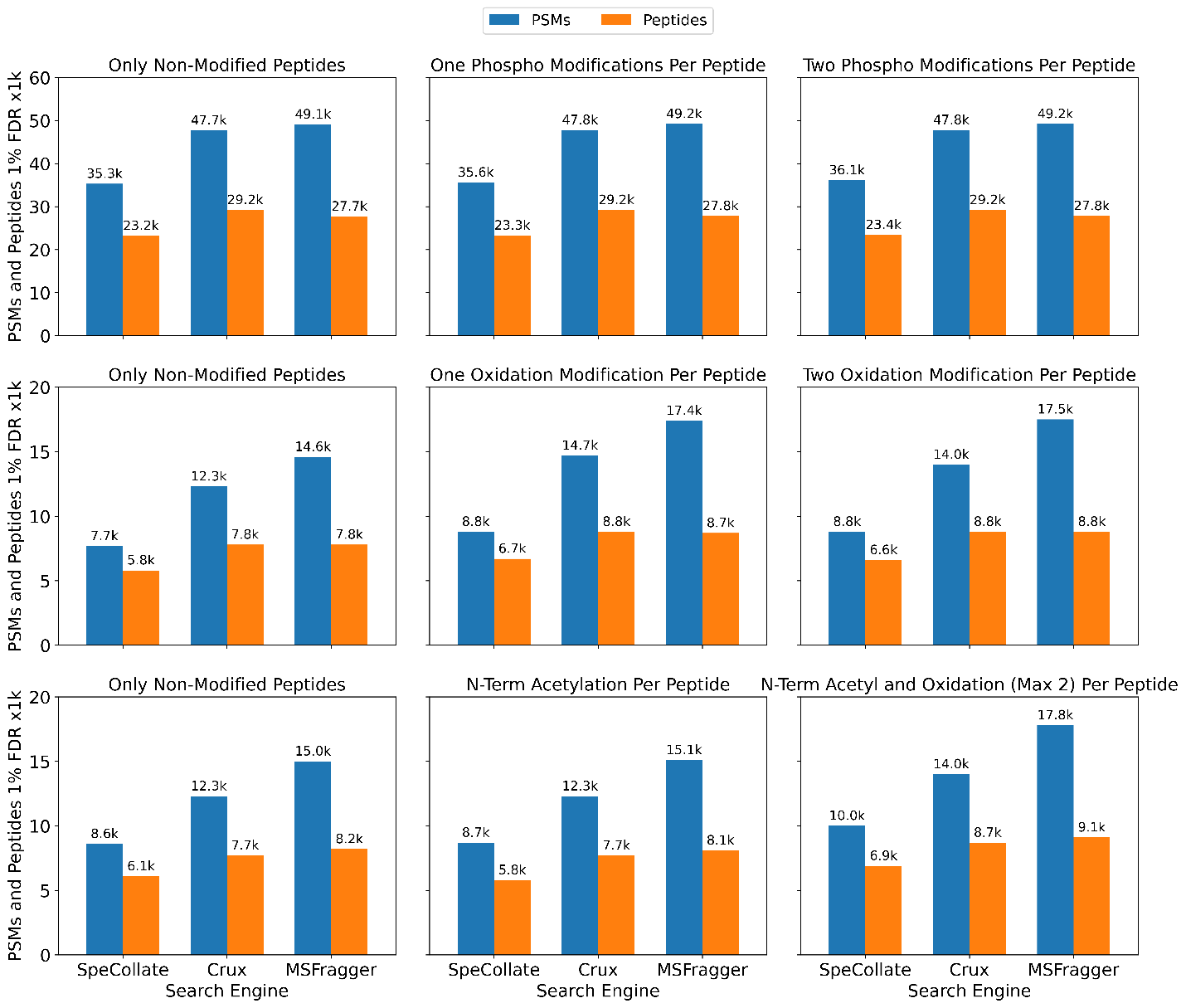


PXD001468

PXD009861

PXD000612
